# From Damage to Dysregulation: Reframing Oxidative Stress and Redox Homeostasis in Central Nervous System Disease

**DOI:** 10.3390/ijms27188374

**Published:** 2026-09-20

**Authors:** Leonard A. Lado

**Affiliations:** Lado Healing Institute, 9410 Fountain Medical Ct, Suite 200, Bonita Springs, FL 34135, USA; heal@ladomd.com

**Keywords:** redox homeostasis, oxidative stress, clinical staging, neurodegeneration, glutathione, biomarkers, clinical trial design, oxidative eustress, neuropsychiatric disease, redox signaling

## Abstract

Antioxidant compounds have repeatedly failed to modify the course of neurodegenerative and psychiatric disease, and the field concluded that oxidative stress is a consequence rather than a driver of neurodegeneration. This paper advances the hypothesis that the error was translational rather than conceptual, and that oxidative damage, redox state, regulatory reserve, disease activity, and accumulated injury are distinct biological dimensions that have been treated as interchangeable in trial design. Oxidative stress was defined from the outset as a disturbance of a regulated balance, and later as a disruption of control across thiol/disulfide couples—paired reduced and oxidized species whose ratios set the local redox state—that are not mutually equilibrated. It was nonetheless investigated as accumulated damage and treated by subtraction: fixed doses of non-selective compounds, given to patients enrolled on clinical diagnosis, without prospective characterization of redox state or regulatory capacity. Four failure modes follow: wrong stage, wrong compound, wrong readout, wrong population. We engage the strongest counterevidence directly, including two trials that corrected compound selectivity and molecular stratification and still returned null results, define redox reserve operationally, propose a four-domain characterization framework for which no cut-points are yet established, and specify the conditions under which the hypothesis should be abandoned.

## 1. Introduction

Few hypotheses in modern neuroscience have been so widely accepted and so therapeutically unproductive as the proposition that oxidative stress contributes to neurodegeneration. The observation that reactive oxygen species damage lipids, proteins, and nucleic acids is now seven decades old [1,2]. Elevated markers of oxidative damage have been documented across several neurodegenerative disorders, though not uniformly [3,4]. The brain’s vulnerability is not in dispute: high oxygen consumption, abundant polyunsaturated lipids, and modest antioxidant reserve make it a plausible target.

And yet the therapeutic record is close to empty. Large randomized trials of antioxidant compounds in neurodegenerative disease have, with few exceptions, failed to modify clinical trajectories [5,6,7,8]. The interpretive consensus that followed was that oxidative damage is a consequence of neurodegeneration rather than a driver of it—an epiphenomenon downstream of whatever the real pathology turns out to be.

This paper argues that the consensus rests on a category error, and that the error is one of investigation rather than of theory.

Oxidative stress was defined from the outset as a disturbance of a regulated balance. Sies and Cadenas described their methods as providing insight into the pro-oxidant–antioxidant balance of the intact cell or organ [9], and Sies subsequently stated the definition plainly: oxidative stress results from imbalance in the pro-oxidant–antioxidant equilibrium in favor of the pro-oxidants [10]. Jones later sharpened the account, arguing that the major cellular thiol/disulfide systems are not in redox equilibrium with one another and that oxidative stress is therefore better understood as a disruption of redox signaling and control [11]. The concept has been homeostatic for forty years.

One consequence of that framing deserves to be stated at the outset. The body already possesses an inducible antioxidant system under transcriptional control, with a sensor, an effector arm, and feedback (Section 3). Every trial considered here supplied antioxidant capacity from outside that system without measuring whether the system itself was intact, failing, or overwhelmed.

Oxidative stress has nonetheless been *investigated* as damage. Oxidative stress has been operationalized as an accumulated quantity, detected after the fact in post-mortem tissue or peripheral fluid, and treated by subtraction—administer an antioxidant, expect less damage. That approach is coherent only if oxidative stress is a scalar with a monotonic dose–response and an optimum at zero. It is none of those things.

**Figure 1:**
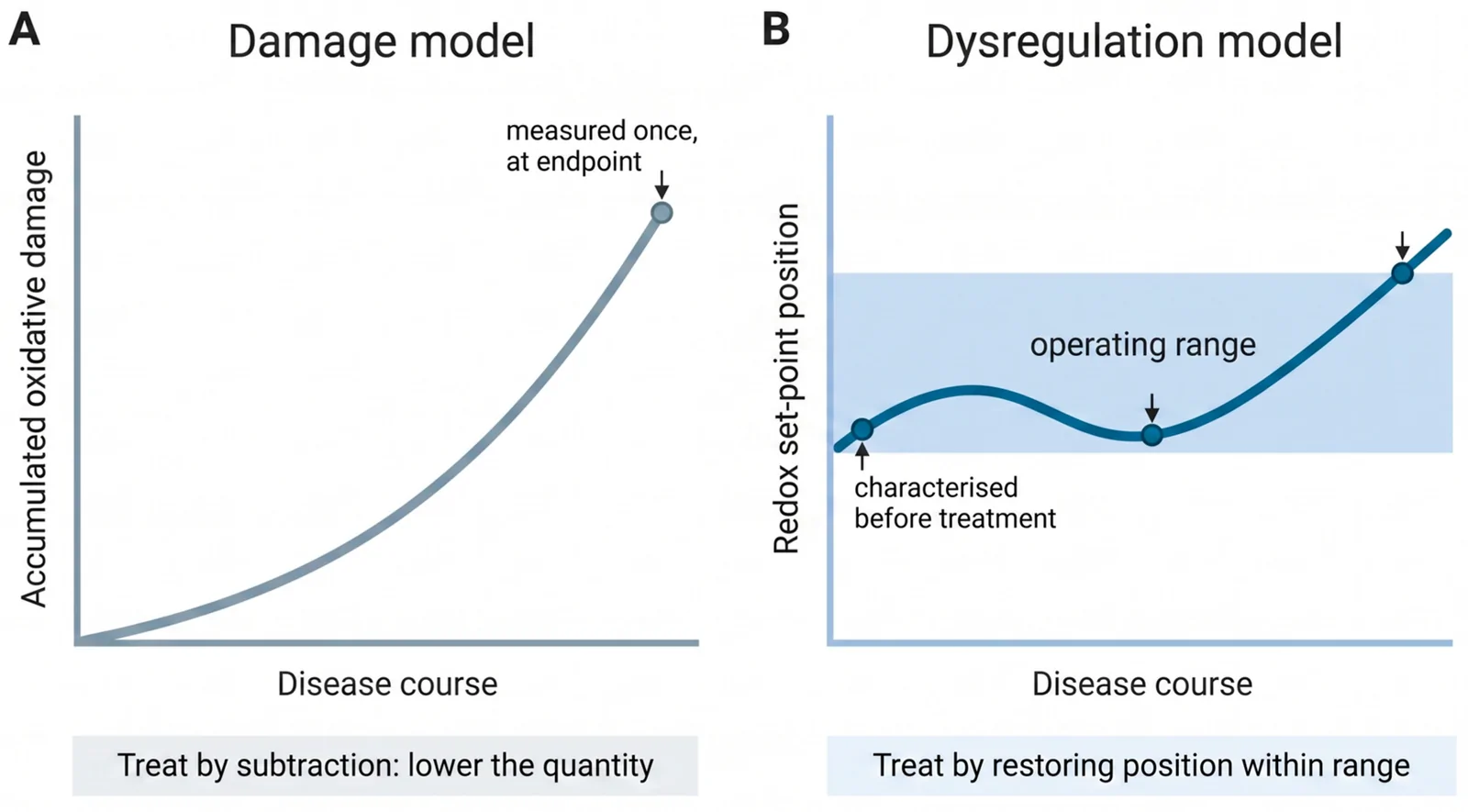
Two models of oxidative stress in central nervous system disease. (**A**) Under a damage model, oxidative stress is an accumulating quantity, measured once at the study endpoint and treated by subtraction. (**B**) Under a dysregulation model, the quantity of interest is the position of a regulated variable relative to its operating range, which must be characterized in the individual before treatment. The two models imply different measurements, different enrollment criteria, and different timing of intervention. Created in BioRender. Lado, L. https://BioRender.com/wll9jmh (accessed on 29 August 2026).

The distinction is not semantic, because damage and dysregulation imply different experiments (Figure 1). A damage model asks how much has accumulated, and answers it at the end. A dysregulation model asks where a regulated variable sits relative to its operating range, and requires that the question be answered *before* treatment, in the individual patient, in the compartment of interest. It changes what is measured, whom one enrolls, and when one intervenes.

Three developments make the reframe timely. First, redox biology has been substantially revised: reactive oxygen species (ROS) are now understood as physiological signaling intermediates operating within a narrow concentration range [12,13,14]. Indiscriminate scavenging—supplying a compound that reacts with and neutralizes reactive species wherever it encounters them, without regard to which species, in which compartment, or to what end—was therefore never a coherent intervention: it disrupted signaling as readily as it removed damage. Second, the trials that defined the field share a set of structural weaknesses that are visible in retrospect and remediable in prospect. Third, and most consequentially, fluid and imaging biomarkers now exist that permit the redox state to be characterized prospectively rather than inferred retrospectively.

We take these in turn. Section 2 traces the conceptual history in three eras. Section 3 sets out the endogenous antioxidant system—the inducible, regulated defense the body already possesses—and the distinct ways it fails, because the trials supplemented that system from outside without ever assessing whether it was working. Section 4 examines the trial failures systematically, identifying their common root and four distinct expressions of it. Section 5 assesses what current biomarkers can and cannot support. Section 6 argues that the missing instrument is a staging framework—the tool oncology uses routinely and neurology has adopted only partially—and sets out what one built on redox principles would require. Section 7 addresses the structural obstacles—methodological, regulatory, and economic—that stand in the way.

The argument is not merely hypothetical. One free radical scavenger has already failed in an unstratified population and succeeded, in the same disease, when enrollment was restricted to patients characterized before treatment—edaravone in amyotrophic lateral sclerosis [15]. A second approved neurological drug, omaveloxolone in Friedreich ataxia, acts by activating the Nrf2 pathway rather than by scavenging [16]. Neither validates redox-based stratification, and we say so at the outset: edaravone’s enrichment criterion was clinical [15], and omaveloxolone’s population was defined by genotype [16]. What they illustrate is the value of defined populations, and Section 6.2 sets out why that is a proof of principle for process- and mechanism-based enrollment rather than for the redox stratification this paper proposes.

Those two facts set the practical stakes of the question. If a compound can fail in one population and succeed in another without the compound changing, then the trial record does not tell us which compounds are ineffective—it tells us which were given to unselected patients. Some of what has been tested and abandoned may work in the right patients, and identifying them requires measurement rather than new chemistry. Whether that is so is an empirical question, and Section 5 and Section 6 set out what would have to be measured to answer it. Both are examined in Section 6. They indicate that the redesign proposed here has already been performed twice, without being described in these terms.

We are not alone in reaching the general conclusion. Day has recently argued for the lung that decades of antioxidant trials failed not because redox biology is invalid but because of conceptual and design flaws in drug development, that indiscriminate suppression of reactive species ignores their signaling roles and spatiotemporal compartmentalization, and that a precision redox medicine framework with biomarker-driven stratification is required [17]. That argument was developed independently, in a different organ system, from a different literature. We take the convergence as corroboration rather than as competition, and note in Section 7.5 that it is itself consistent with the proposition that the underlying chemistry is not tissue-specific.

What is advanced here beyond that shared reframe is threefold: a distinction between resting redox concentration and regulatory reserve, with an operational definition and a proposed challenge protocol (Section 3.3); a four-domain characterization framework separating reserve, displacement, activity, and accumulated injury (Section 6.3); and explicit conditions under which the hypothesis should be abandoned (Section 4.7 and Section 6.4).

The molecular basis of the argument should be stated before it is made. Cellular redox state is not a scalar quantity but the position of specific thiol/disulfide couples—reduced and oxidized glutathione, the thioredoxin system, cysteine and cystine—which are compartmentalized and not in equilibrium with one another [11]. Those couples are held within operating ranges by an inducible control system in which Kelch-like ECH-associated protein 1 (KEAP1) acts as a cysteine-based sensor and nuclear factor erythroid 2-related factor 2 (NRF2) as the transcriptional effector, driving glutathione synthesis at glutamate cysteine ligase and the thioredoxin and peroxiredoxin systems [18]. Hydrogen peroxide within this arrangement is a signaling intermediate at low nanomolar concentrations and damaging only well above them [13]. Section 3 develops this machinery in full; the question this paper asks is whether any of it was measured in the patients whose trials are said to have tested it.

**Thehypothesis may be stated directly.** Five different things can be true of a patient with a neurodegenerative or psychiatric disorder, and they are not the same thing: how much oxidative damage has already accumulated in their tissue, where their redox couples currently sit relative to the range those couples normally occupy, how much capacity they retain to restore that position when challenged, whether the disease process is active at this moment and how fast it is moving, and how much irreversible injury has been sustained. A patient may have extensive accumulated damage and an intact regulatory system, or a normal resting measurement and no reserve at all. These are five distinct clinical facts, and none of them predicts the others. Two of these, accumulated oxidative damage and irreversible injury, are both consequences already sustained, and neither says anything about how the regulatory system is functioning; Section 6.3 therefore records them on a single axis, which is why five clinical facts become four axes there.

The trials treated them as one. Patients were enrolled on a diagnosis, given the same compound at the same dose irrespective of any of these five, and followed to a clinical endpoint. The comparison is not to a trial of a drug that failed; it is to a trial in which every hypertensive patient received the same antihypertensive at the same dose without anyone measuring blood pressure, and the field concluded from the null result that blood pressure is not causally related to stroke.

If that is the right reading, then what the null results establish is narrow: indiscriminate antioxidant supplementation does not modify the course of clinically diagnosed neurodegenerative disease. They do not establish that redox dysregulation is causally unimportant in patients selected on the basis of their actual redox state, or treated earlier in the course, or given a compound directed at the specific abnormality they have. The hypothesis is testable, and Section 4.7 and Section 6.4 specify the conditions under which it should be abandoned.

Our claim is therefore deliberately narrow. We do not argue that oxidative stress is the primary cause of any neurodegenerative or psychiatric disorder. We argue that the existing evidence is insufficient to reject that possibility, because the trials that would have tested it were built on a model of the phenomenon that the underlying biology had already abandoned.

## 2. Three Eras

### 2.1. Origins: Oxygen as Poison, 1954–1990s

The modern free radical hypothesis has a precise origin. In 1954, Gerschman and colleagues at Rochester reported that oxygen poisoning and X-irradiation shared a common mechanism, proposing free radical formation as the unifying agent of injury [1]. Two years later, Harman extended the argument from acute toxicity to the biology of aging itself, suggesting that cumulative free radical damage accounted for senescence [2].

The framing established in those two papers proved durable and, in one respect, limiting. Free radicals entered biology as agents of damage. The conceptual vocabulary that followed—oxidative *stress*, oxidative *injury*, antioxidant *defense*—encoded a military metaphor in which reactive species were the aggressor and antioxidants the protective response. Within that frame, the therapeutic inference was immediate and apparently obvious: if reactive species cause damage, more antioxidants should mean less damage.

This is the frame that persisted into trial design, and it is worth being precise about what it displaced. The homeostatic account was not a later correction to the damage account; the two developed in parallel, and the homeostatic one was the formal definition [9,10]. What the damage metaphor supplied was not a theory but an intuition—one that translated directly into a dosing schedule, which the homeostatic account did not.

Through the 1980s and 1990s, the intuition was reinforced by accumulating descriptive evidence. Post-mortem studies documented elevated markers of lipid peroxidation, oxidative DNA damage, and protein oxidation in affected brain regions [3,4].

Two features of that literature are worth recovering, because both were available at the time and neither shaped the trials that followed. The first is that the finding was not universal: Alam, Halliwell, and Jenner applied to Huntington’s disease the same methods that had demonstrated increased oxidative damage in Parkinson’s disease, Alzheimer’s disease, and senile dementia of the Lewy body type, and found no elevation in lipid peroxidation, oxidative DNA damage, or protein carbonyls in caudate, putamen, or frontal cortex [4]. Oxidative damage was a feature of some neurodegenerative disorders and not others—a pattern consistent with the possibility, revisited in Section 4.6, that the hypothesis is narrower than its general statement implies.

The second is more directly damaging to the causal reading. In Parkinson’s disease, protein carbonyls were elevated not only in substantia nigra, caudate, and putamen but also in regions not thought to be affected, raising the possibility of a contribution from L-DOPA therapy; brain regions from individuals with incidental Lewy body disease—putative presymptomatic Parkinson’s disease—showed no rise in carbonyls anywhere [3]. The authors concluded that oxidative protein damage occurs widely but late and/or that treatment contributes to it [3].

That is a finding of considerable importance to the present argument, and it cuts in a specific direction. It does not show that redox dysregulation is absent early; it shows that *accumulated damage* is absent early. A marker that is normal in presymptomatic disease and elevated in treated, established disease is behaving as a trailing indicator, which is precisely what Section 7.1 argues damage products are. The correlational case for oxidative damage was therefore strong, and the causal case was assumed rather than tested—and the intervention that followed from the assumption was scavenging.

### 2.2. The Trial Era: 1993–2014

Three trials illustrate the pattern and its outcome.

**DATATOP(1993).** The Parkinson Study Group randomized 800 patients with early, untreated Parkinson’s disease to deprenyl, alpha-tocopherol, both, or placebo, with onset of disability requiring levodopa as the primary endpoint. Tocopherol at 2000 IU daily produced no benefit and no interaction with deprenyl. Deprenyl delayed the endpoint (hazard ratio 0.50, 95% CI 0.41–0.62, *p* < 0.001)—that is, at any given moment, a patient on deprenyl was about half as likely to reach the point of requiring levodopa as one on placebo, and the confidence interval excludes any possibility that the effect was trivial. The authors were nonetheless explicit that its mechanism remained unclear and its early, partly reversible profile was consistent with symptomatic rather than protective action [5].

The internal contrast is the useful part. Both arms were tested in the same 800 patients, by the same investigators, against the same endpoint. One arm produced a halving of risk; the other produced nothing. Whatever else may be said about the tocopherol result, it cannot be attributed to a trial too small or too insensitive to detect an effect, because the trial detected a large one in the adjacent arm. The null is real, and it therefore requires an explanation of the kind Section 4.2, Section 4.3, Section 4.4 and Section 4.5 set out, rather than an appeal to inadequate power.

**The Alzheimer’s Disease Cooperative Study trial (1997).** Sano and colleagues randomized 341 patients with moderate Alzheimer’s disease to selegiline, alpha-tocopherol, both, or placebo over two years. This trial is frequently remembered as positive, and the detail of how it became positive matters. In the unadjusted analysis, there was no statistically significant difference among the four groups. Significance emerged only after adjustment for baseline Mini-Mental State Examination score, which was imbalanced in favor of placebo despite randomization and was itself highly predictive of outcome [6]. A result contingent on post hoc covariate adjustment for a baseline imbalance is weak evidence on which to found a therapeutic era.

**QE3 (2014).** Beal and colleagues randomized 600 patients with early Parkinson’s disease to placebo, 1200 mg/day, or 2400 mg/day coenzyme Q10, and terminated the trial on a prespecified futility criterion. Both active arms showed slight adverse trends: adjusted worsening on the total Unified Parkinson’s Disease Rating Scale (UPDRS) was 6.9 points on placebo, 7.5 at 1200 mg, and 8.0 at 2400 mg [7]. All participants, including placebo, received 1200 IU/day of vitamin E, which complicates any inference about antioxidant exposure as such.

**TEAM-AD (2014).** One trial in this series reported benefit, and it belongs in the record. Dysken and colleagues randomized 613 patients with mild to moderate Alzheimer’s disease, all receiving a cholinesterase inhibitor, to alpha-tocopherol 2000 IU/day, memantine 20 mg/day, both, or placebo, with change on the Alzheimer’s Disease Cooperative Study Activities of Daily Living Inventory as the primary outcome. Over a mean follow-up of 2.27 years, functional decline in the alpha-tocopherol arm was 3.15 units less than in the placebo arm (95% CI 0.92 to 5.39; adjusted *p* = 0.03), a 19% slower rate of progression per year; memantine alone and the combination showed no significant benefit [8]. The result meets a standard the 1997 trial did not: it was the prespecified primary endpoint, in the primary analysis, with a confidence interval that excludes zero, rather than a finding recovered by post hoc adjustment. Its scope should also be stated precisely. The ADCS-ADL Inventory is a caregiver-rated measure of everyday activities, and it was on this measure that decline was slower; the cognitive scales collected as secondary outcomes did not separate from placebo. Patients on alpha-tocopherol lost function more slowly. They did not think or remember better. It was obtained at the same fixed dose as the trials above, in a population enrolled on clinical diagnosis, without any redox measurement, and it therefore does not resolve the question this paper asks: a positive result under those conditions is no more interpretable about mechanism than the nulls that surround it. What it does establish is that the record is not uniform, and the statements that follow are qualified accordingly.

By 2014, the cumulative record supported a reading that was nearly, though not entirely, uniform: with the TEAM-AD functional result as the exception [8], antioxidant supplementation had not modified neurodegenerative disease. The inference commonly drawn from that reading was broader—that oxidative stress is not causally upstream. That broader inference does not follow, and Section 4 examines why.

### 2.3. The Signaling Correction: 2000s–Present

While the trials were running, the underlying biology was being substantially revised.

The reframing is captured most directly by Finkel, who set out the case that reactive oxygen species function as physiological regulators of intracellular signaling rather than as purely harmful byproducts, acting principally through reversible covalent modification of redox-sensitive cysteine residues in target proteins [12]. On this account, ROS are not noise in the system. They are part of the signal.

Sies formalized the concentration dependence. Hydrogen peroxide in the low nanomolar range (approximately 1–10 nM) operates in redox sensing and regulation—termed oxidative *eustress*. Higher concentrations engage adaptive stress responses through master switches, including Nrf2–Keap1 and NF-κB. Only at supraphysiological concentrations above roughly 100 nM does damage to biomolecules predominate, termed oxidative *distress* [13].

**Figure 2:**
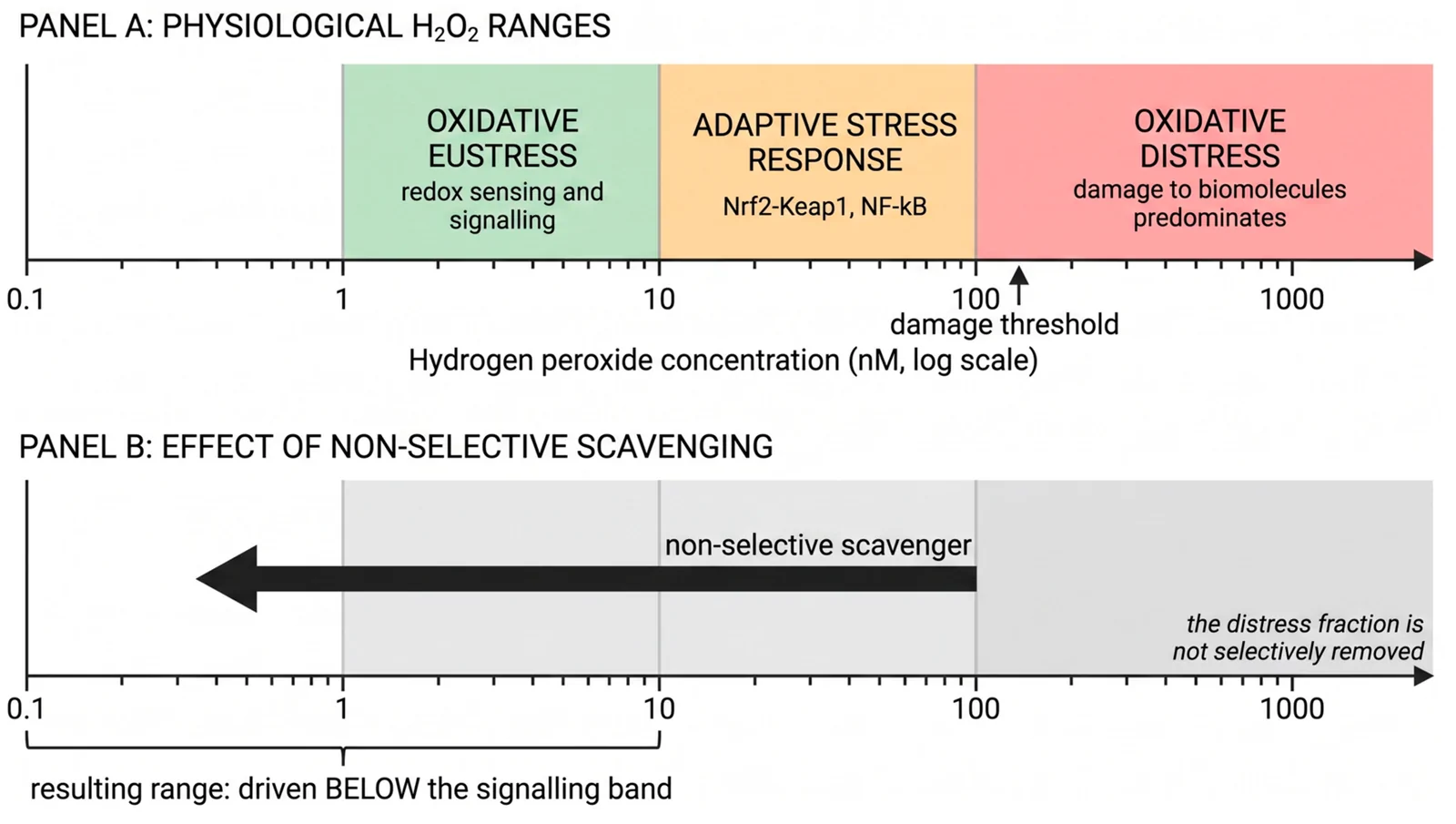
Concentration dependence of hydrogen peroxide and the consequence of non-selective scavenging. (**A**) H_2_O_2_ in the low nanomolar range supports redox sensing and signaling (oxidative eustress); higher concentrations engage adaptive stress responses through Nrf2–Keap1 and NF-κB; damage to biomolecules predominates only above approximately 100 nM (oxidative distress) [13]. (**B**) A scavenger acting without regard to species, compartment, or concentration lowers the entire range rather than removing the distress fraction selectively, driving the regulated variable below the signaling band on which growth factor signaling and stress adaptation depend. The bracket in Panel B marks the resulting range, which extends below the lower bound of the signaling band. Created in BioRender. Lado, L. https://BioRender.com/8irre1z (accessed on 29 August 2026).

The therapeutic implication is severe and, in retrospect, was available before most of the trials reported (Figure 2). An intervention that lowers reactive species indiscriminately does not selectively remove the distress component. It compresses the whole range, including the eustress band on which growth factor signaling, stress adaptation, and inflammatory regulation depend. Gram-quantity dosing of a non-selective scavenger is not a targeted intervention against a pathological process; it is a perturbation of a signaling system with a therapeutic window that was never characterized.

Sies and Jones have since stated the consequence for drug development explicitly: nonspecific elimination of reactive species by low-molecular-mass antioxidant compounds did not counteract disease initiation or progression in clinical trials, and selective targeting of specific ROS-mediated signaling pathways represents the more defensible path forward [14].

That conclusion is where this paper starts, not where it ends. The general principle—that selectivity should replace indiscriminate scavenging—has been stated. The specific work for the nervous system has not. Three questions remain open. Which particular failures account for the trial record? Which of those can current biomarkers actually detect in a living patient? And what would a trial built to avoid them look like in practice? Section 3, Section 4, Section 5 and Section 6 take these questions in turn.

## 3. The Endogenous System and Its Modes of Failure

### 3.1. The Defense That Is Already Present

The trials of Section 2 supplied antioxidant capacity from the outside. They did so into a system that already possesses an inducible antioxidant apparatus of considerable sophistication, and the relationship between the two has rarely been made explicit.

That apparatus is not a stockpile of scavengers. It is a regulated control system with a sensor, an effector arm, and a feedback loop. NRF2 is an intracellular protein and a transcription factor; its function is to bind DNA and switch genes on. It is not secreted, has no extracellular form, and is not a receptor—nothing outside the cell interacts with it. What changes in the course of the events described below is not whether NRF2 is present but *which compartment of the cell it occupies*, and that distinction carries the argument of Section 3.3.

**Figure 3:**
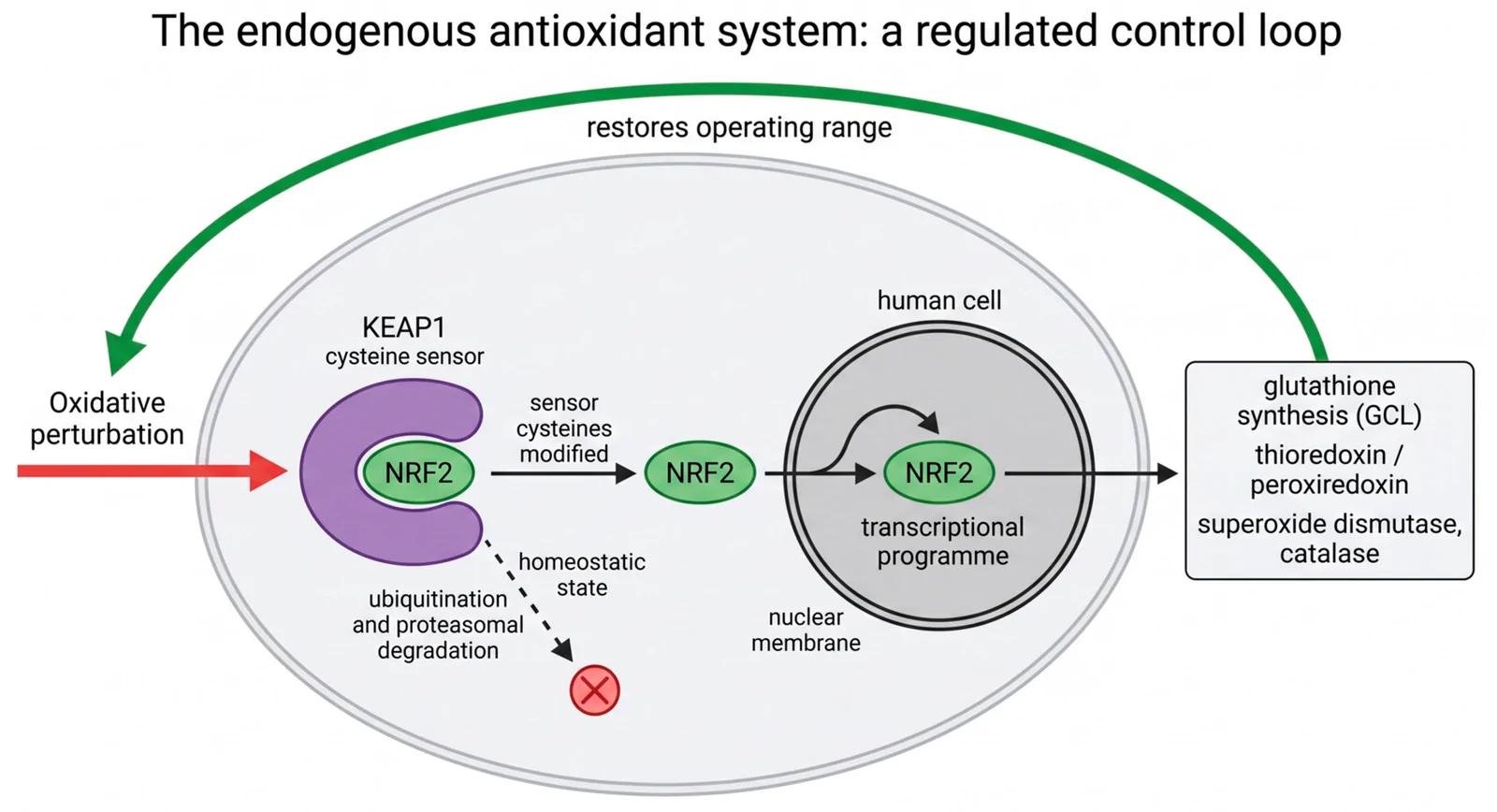
The endogenous antioxidant system as a regulated control loop. Under homeostatic conditions, KEAP1 binds NRF2 and directs it to ubiquitination and proteasomal degradation (dashed arrow; the encircled cross marks NRF2 destroyed, so the antioxidant program remains off). Modification of KEAP1 sensor cysteines by oxidative perturbation abolishes its repressor function; NRF2 is stabilized, translocates to the nucleus, and induces a transcriptional program encompassing glutathione synthesis, the thioredoxin and peroxiredoxin systems, and superoxide dismutase and catalase, whose output returns the cell toward its operating range [18]. The system has a sensor, an effector arm, and feedback, and is therefore a homeostatic mechanism rather than a reservoir of scavengers. Created in BioRender. Lado, L. https://BioRender.com/0gbuhwb (accessed on 29 August 2026).

Under homeostatic conditions, NRF2 is held in the cytoplasm and is short-lived, bound avidly by Kelch-like ECH-associated protein 1 (KEAP1), which functions both as a substrate adaptor for a Cullin 3-based E3 ubiquitin ligase that targets NRF2 for ubiquitination and proteasomal degradation and as a cysteine-based sensor for a range of physiological and pharmacological activators [18]. The terms describe the cell’s routine disposal system: ubiquitin is a small protein that cells attach to other proteins as a disposal tag, an E3 ubiquitin ligase is the enzyme complex that attaches it, and the proteasome is the structure that then breaks the tagged protein down. KEAP1 holds NRF2 where that tagging occurs, so under resting conditions NRF2 is continuously marked for destruction and degraded almost as fast as it is made, and the genes it controls stay off. Chemical modification of specific sensor cysteines abolishes the repressor function of KEAP1. NRF2 is then no longer degraded, accumulates in the cytoplasm, and crosses the nuclear membrane into the nucleus, where it binds regulatory DNA sequences and induces large networks of antioxidant, anti-inflammatory, and cytoprotective proteins while suppressing pro-inflammatory mediators [18]. The whole cycle is intracellular: cytoplasm when the system is quiet, nucleus when it is activated. The pathway is shown in Figure 3.

Downstream of that switch sit the effectors familiar from the descriptive literature: glutamate-cysteine ligase and the glutathione system, the thioredoxin and peroxiredoxin couples, superoxide dismutase, and catalase. These are the components usually listed when the endogenous defense is described, and listing them is exactly what obscures the point that matters here: the system is inducible and regulated, not a stockpile.

Because the argument that follows depends on it, one term should be defined before it is used further. A **redox couple** is a pair of chemically related species—a reduced form and its oxidized counterpart—whose ratio sets the local redox state. Reduced and oxidized glutathione (GSH/GSSG) is the most familiar example; reduced and oxidized thioredoxin, and cysteine and cystine, are others. The ratio, rather than the absolute amount of either species, is what a cell regulates and what determines whether a redox-sensitive protein is switched on or off.

Two features of these couples matter for everything that follows. They are **compartmentalized**: the GSH/GSSG ratio in mitochondria differs from that in the cytosol and in the extracellular space, within the same cell at the same moment. And they are **not mutually equilibrated** [11]—knowing where one couple sits does not tell you where another sits, even in the same compartment. It follows that there is no single number that describes “the” redox state of a patient, a tissue, or a cell, which is why Section 4.1 argues that a design premised on lowering one quantity was mismatched to the biology from the outset.

One further term follows from this and is used throughout. A couple is **displaced** when its ratio has moved outside the range within which it normally operates—for example, when the GSH/GSSG ratio in a given compartment falls below the range that cell type sustains under ordinary conditions. Displacement is a statement about position relative to a range rather than about absolute concentration, and it has a direction as well as a magnitude: a couple may be displaced toward oxidation or toward reduction, and the two are not equivalent, since the signaling functions described in Section 2.3 are lost at the reduced extreme just as damage predominates at the oxidized one. The system senses a perturbation, mounts a graded response, and returns the cell toward its operating range, which is the definition of a homeostatic mechanism and precisely the object that Section 4 argues the trials never measured.

### 3.2. Why This Reframes the Trial Record

The consequence for interpretation is direct, and it is mechanistic rather than procedural. It is easiest to see if the causal architecture is stated in order:

redox dysregulation → altered redox signaling and adaptive response → cellular dysfunction → oxidative molecular damage → downstream neuronal injury

Damage products sit in the fourth position in that sequence. This is not an argument that they are the wrong measurements; they are accurate measurements of what they measure. It is an argument that they are downstream, and that a downstream measurement cannot by itself establish whether an upstream regulatory abnormality is causal, compensatory, or epiphenomenal. That determination requires measurement at the first position, which is where the trials were silent.

If a patient’s endogenous system is intact and responding, exogenous antioxidant is largely redundant: the cell is already doing, under regulation, what the supplement does without regulation. If the endogenous system has failed, exogenous antioxidant does not repair it. It substitutes for a fraction of the system’s output while leaving the control loop itself broken—and, as Section 4.3 sets out, a non-selective scavenger simultaneously compresses the signaling range on which the regulator depends for its own activation [13,14].

Either way, supplementation is the wrong lever. This is a stronger statement than the four failure modes cataloged in Section 4, which explain why the trials could not have detected an effect. The present argument explains why there may have been no effect to detect: the intervention was not directed at the system that determines redox state.

The parallel with endocrine practice is instructive, provided it is not pressed too far. Thyroid hormone is given to patients in whom the regulatory axis has been shown to have failed, at a dose titrated against a measured feedback variable, and it is not given to patients whose axis is intact. The antioxidant trials did the opposite on all three counts.

### 3.3. How the System Fails, and Why the Answer Differs by Disorder

The most consequential observation for the present hypothesis is that endogenous failure is not a single phenomenon. Ramsey and colleagues examined NRF2 localization in affected brain regions across neurodegenerative disorders and found opposite patterns [19].

In the normal hippocampus, NRF2 was present in both the nucleus and cytoplasm of neurons, predominantly nuclear. In Alzheimer’s disease and the Lewy body variant, NRF2 was predominantly *cytoplasmic* in hippocampal neurons, with a significant decrease in nuclear NRF2 on immunoblotting. The authors’ interpretation is the important one: NRF2-mediated transcription is not induced in Alzheimer’s neurons *despite the presence of oxidative stress* [19]. The sensor is not translating a perturbation into a response.

In Parkinson’s disease, the finding inverts. NRF2 was strongly nuclear in nigral neurons—the response is induced—but the authors conclude it may nonetheless be insufficient to protect those neurons from degeneration [19]. Signaling is intact; capacity is exceeded.

A third pattern appears in schizophrenia, and it is constitutional rather than acquired. The deficit demonstrated by Gysin and colleagues emerged when fibroblasts were challenged: glutamate cysteine ligase activity fell 26% below controls, correlating with reduced GCLC expression, and a *GCLC* trinucleotide repeat polymorphism associated with the disorder tracked lower enzyme activity and lower glutathione content [20]. Here the switch may operate normally while the effector arm has a genetically constrained ceiling.

A distinction follows from the third pattern that applies to all three, and it is central to what this paper proposes. The deficit demonstrated in schizophrenia appeared *under* *challenge*; at rest, a compensated system may hold its regulated variable within range precisely because regulation is working. **Resting redox concentration is therefore not the same quantity as regulatory reserve.** A system can present a normal resting value while having a markedly reduced capacity to restore that value when perturbed, and the two dissociate exactly where it matters most—in the individuals who are compensating rather than in the ones who have already failed.

We define the term operationally. **Redox reserve** is the capacity of a biological system to maintain or restore a compartment-specific redox variable within its physiological operating range following a standardized perturbation. It is a property of the individual rather than of the disorder, and it is not determined by genotype alone: synthetic capacity set by the glutathione-synthesis genes is one contributor, but hormonal status, the developmental stage at which an insult is sustained, and accumulated environmental load are other contributors. Two individuals carrying the same oxidative burden but differing in reserve are not in the same position, and no measurement of that burden distinguishes them.

The analogy is to glucose tolerance rather than to fasting glucose: fasting glucose can be normal in a person whose regulatory capacity is substantially impaired, and the abnormality emerges only under a controlled load. The relationship of this term to established redox concepts should be made explicit. The eustress/distress framework specifies where a regulated variable sits relative to the concentration band in which it signals [13]; the redox control model specifies that each thiol/disulfide couple is held in position by its own regulatory machinery rather than by a global equilibrium [11]. Both are descriptions of position. Reserve is a property of the control loop itself: how far it can be displaced and still return, and how quickly. It adds a dynamic quantity to two static ones, and it is the quantity that a trial enrolling on resting values cannot see. In practical terms, the assessment would follow the bioenergetic template set out below: cells or a compartment are exposed to a graded, standardized oxidative load, the displacement and recovery of a defined couple (GSH/GSSG in the first instance) are followed over a defined window, and reserve is expressed as the magnitude of load the system can absorb while still restoring its operating range. Peripheral blood mononuclear cells or cultured fibroblasts challenged ex vivo with a titrated oxidant are the nearest available implementation and could be validated with existing methods; an in vivo version pairing a physiological perturbation with regional glutathione MRS before and after is conceivable but further off. Neither is validated, and Section 5.2 treats the absence of such a protocol as the principal gap.

**Two distinctions are necessary here, because neither the idea of reserve nor its measurement under challenge is new.** The first is total antioxidant capacity, which is measured routinely in plasma and which a reader may reasonably suppose already provides what we are asking for. It does not, for two reasons. It is a summed scavenging measure that does not resolve which couple is displaced or in which compartment, and it is a static quantity rather than a response to perturbation. Its behavior in disease illustrates the difficulty directly: in pregnant women with COVID-19, total antioxidant capacity was *higher* in severe than in non-severe disease, and the authors interpreted the elevation as a counterregulatory response rather than as preserved capacity [21]. A measure that rises with severity because the system is straining cannot distinguish a patient with reserve from one who is compensating hard and about to fail. That distinction is precisely what the R axis is for.

The second distinction concerns whether any of this is measurable. The objection to be answered is that we are asking for a test nobody has. In fact, a test of exactly this kind already exists, for a different variable.

It is called bioenergetic reserve. Every cell consumes oxygen at rest but can consume considerably more when required; the difference between the two is its spare capacity. The assay measures it directly. A chemical uncoupler is applied to force the mitochondria to their maximum, and the increase above resting consumption is recorded. The result is a number describing how much the cell had in hand.

Two features of that assay matter here. It works in cells drawn from living patients—peripheral blood mononuclear cells obtained by venipuncture—and it is sensitive enough to detect change: in one study, spare respiratory capacity in such cells rose 5.65-fold over twelve weeks of aerobic exercise [22].

What we propose for redox reserve is the same procedure applied to a different quantity. Perturb the system, measure what it restores, express the difference as capacity. The existence of a working assay for bioenergetic reserve is therefore the strongest available evidence that the redox equivalent is achievable rather than merely desirable. No validated challenge protocol of this kind exists for central redox measurement in humans, and its absence is, in our assessment, the principal methodological gap in the field—a research priority rather than an available test. Section 5.2 returns to it against the negative resting measurement in schizophrenia.

**Figure 4:**
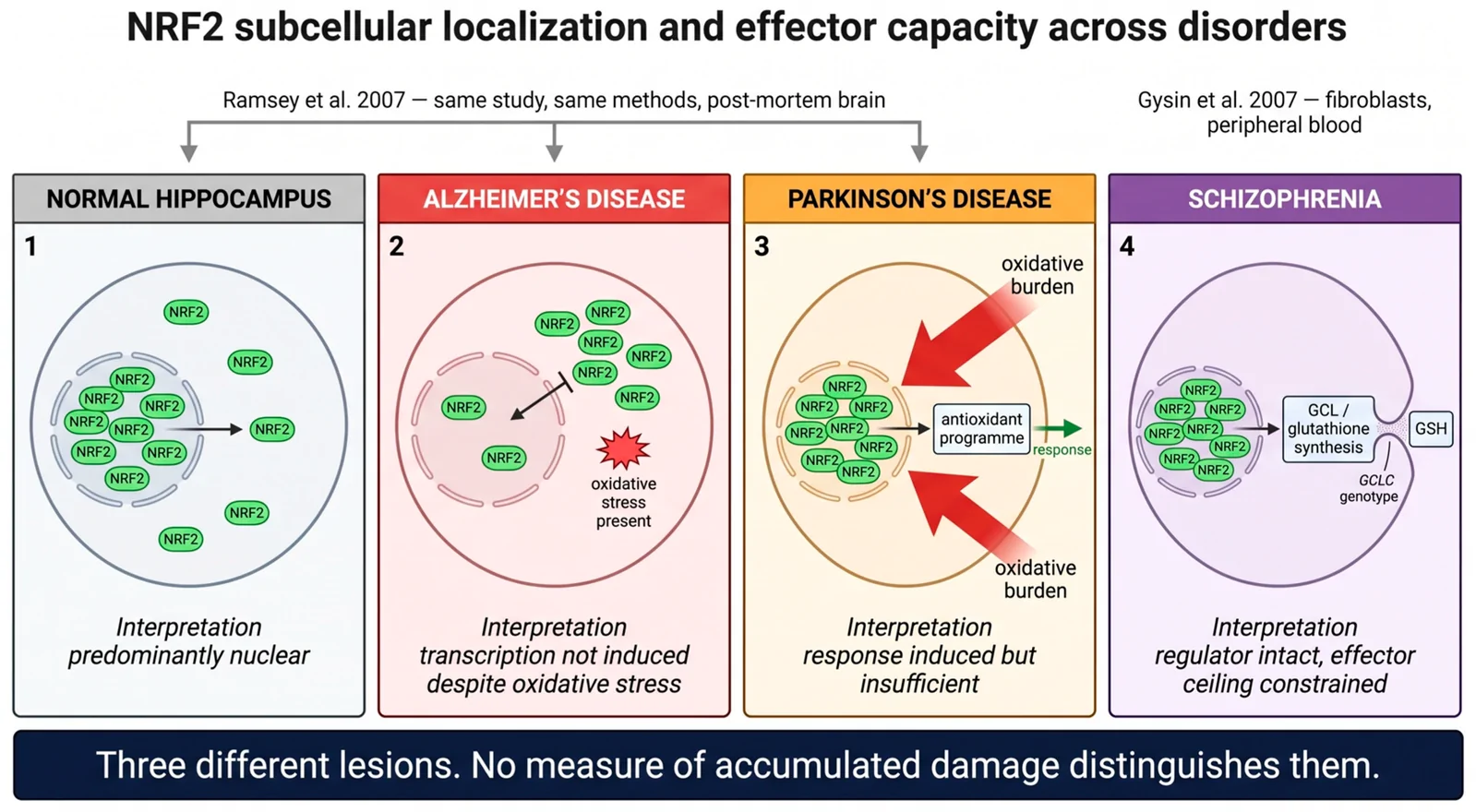
NRF2 subcellular localization and effector capacity across disorders. In normal hippocampal neurons, NRF2 is present in both compartments and predominantly nuclear. In Alzheimer’s disease, it is predominantly cytoplasmic, with a significant reduction in nuclear NRF2, and transcription is not induced despite the presence of oxidative stress—the sensor does not translate a perturbation into a response. In Parkinson’s disease, the pattern inverts: NRF2 is strongly nuclear in nigral neurons, and the response is induced, but may be insufficient to protect them from degeneration. Panels **1**–**3** are from a single study of post-mortem brain, so that contrast is a within-study comparison [19]. In schizophrenia, the regulator may operate normally while the effector arm is constrained: glutamate cysteine ligase activity falls below control values under oxidative challenge, tracking reduced GCLC expression and a disorder-associated *GCLC* trinucleotide repeat polymorphism [20]. Panel **4** is from separate work in cultured fibroblasts and peripheral blood, as labeled; the cell outline is used for visual consistency and does not assert a neuronal localization finding in schizophrenia, and Section 5.3 sets out why peripheral-to-central correspondence cannot presently be assumed. The three lesions imply different interventions, and no measurement of accumulated damage distinguishes between them. Created in BioRender. Lado, L. https://BioRender.com/dgfzjzm (accessed on 29 August 2026).

Three patterns, then, and they are not interchangeable (Figure 4). We present them as mechanistic interpretations of the cited findings, each drawn from a single study, and not as established disease classifications.

### 3.4. The Therapeutic Consequence

Table 1 is the argument of this section, because the three modes cannot be addressed by the same drug.

An NRF2 activator should benefit the first mode, where the switch is failing to engage, and might benefit the second, where the response is real but inadequate. It should not be expected to correct the third, in which the transcriptional response is intact, but the enzyme it induces has a constrained ceiling; that requires substrate provision or a different lever entirely.

This distinction is not hypothetical. The one positive randomized result among the disease-modifying trials discussed here that acted on the endogenous system rather than by supplementation is omaveloxolone in Friedreich ataxia, which acts by activating NRF2 [16]. In the phase 2 MOXIe trial, 103 patients were randomized and 82 analyzed. The modified Friedreich’s Ataxia Rating Scale scores higher with worse neurological function, so a fall in score represents improvement. At 48 weeks, patients receiving omaveloxolone had improved by 1.55 ± 0.69 points while those receiving placebo had worsened by 0.85 ± 0.64, a difference of 2.40 ± 0.96 in favor of treatment (*p* = 0.014) [16]. For scale, the placebo group’s own deterioration over that year was 0.85 points, so the treatment difference corresponds to roughly three years of the decline observed in the untreated arm—an internal comparison within one small trial rather than an estimate drawn from natural history. We give the numbers because the effect is modest and the trial small, and the argument does not require it to be otherwise. Omaveloxolone belongs to the cyclic cyanoenone class identified as the most potent NRF2 activators [18]. Its mechanism is one of pathway modulation rather than exogenous supplementation. We state the inference carefully: the clinical success of an NRF2-directed therapy in Friedreich ataxia is mechanistically consistent with the regulatory model proposed here, but it does not establish that NRF2-mediated redox restoration alone accounts for the therapeutic effect, and the pharmacology of omaveloxolone and the biology of Friedreich ataxia are both broader than that inference would require.

The question this raises is whether compounds exist that act at each point, and the answer is uneven in a way that is itself informative.

**Mode 1, sensing and translocation failure—the pattern reported in Alzheimer’s disease (Table 1)—is best served.** NRF2 activators address precisely this lesion, and the class is clinically established: omaveloxolone in Friedreich ataxia [16], and the fumaric acid esters used in multiple sclerosis, whose neuroprotective effect in experimental autoimmune encephalomyelitis is accompanied by upregulation of Nrf2-dependent antioxidative genes alongside reduced Th1 responses [23]. We note the mechanism is not exclusively redox—the fumarates are immunomodulatory as well—and we do not present them as a clean test of the model.

**Mode 2, capacity exceeded—the pattern reported in Parkinson’s disease—is the least well-served, and this may not be a coincidence.** If the regulator is functioning and the response is simply overwhelmed, then amplifying the response further has a low ceiling, and the therapeutic lever is reduction in burden rather than augmentation of defense. It is worth noting that MitoQ in Parkinson’s disease and elamipretide in primary mitochondrial myopathy—the two clear failures examined in Section 4.6—both attempted to augment capacity in disorders where mode 2 plausibly applies [24,25]. That is an explanation for those nulls which is consistent with the present model rather than an evasion of them, though we advance it as a hypothesis and not as an established account.

**Mode 3, a constrained effector ceiling—the pattern reported in schizophrenia—is addressable by a different lever again.** Where glutamate cysteine ligase activity is limited, inducing more of a rate-limiting enzyme is unlikely to help; raising the supply of its substrate may. N-acetylcysteine does this by providing cysteine, the rate-limiting precursor for glutathione synthesis; it does not bypass glutamate cysteine ligase, and it can raise flux only to whatever ceiling the constrained enzyme retains, so whether provision suffices is an empirical question rather than a settled one. It has been trialed in clozapine-resistant schizophrenia at 2 g daily, with peripheral and cortical glutathione concentrations measured as process outcomes rather than assumed [26]—an enrollment and monitoring design closer to what Section 6.4 proposes than most of the trials in Section 2.

This generates a prediction that is specific and has not been tested: in patients whose *GCLC* genotype constrains synthetic capacity, substrate provision should outperform NRF2 activation, whereas in disorders characterized by impaired NRF2 translocation, the reverse should hold. The claim is not that every disorder requires its own compound, which would be unfalsifiable. It is narrower: three mechanistically distinct lesions imply at least three intervention classes, and a trial that does not establish which lesion a participant has will dilute any true effect across patients for whom the compound is mechanistically inappropriate.

Set against the record in Section 2, the pattern is at least suggestive. Every trial that supplemented exogenous antioxidant capacity returned a null result on its primary outcome, with the exception of TEAM-AD, whose functional benefit was obtained at a fixed dose without any redox characterization [8]. The one trial that targeted the endogenous regulatory system succeeded in a population defined by a monogenic lesion. A single success does not establish the superiority of endogenous restoration over supplementation, and we do not claim it does; the observation aligns with the mechanistic argument rather than against it, and it is the pattern that Section 6 builds the reserve (R) axis of the proposed staging framework upon: *reserve* is not a quantity of antioxidant but the integrity of this system.

## 4. The Autopsy: One Error, Four Expressions

### 4.1. One Omission, Four Expressions

The trials reviewed in Section 2 are usually read as a single result: antioxidants do not work in neurodegenerative disease. Read more closely, they exhibit four distinct problems, each independently sufficient to produce a null finding.

It is worth being explicit about what a null finding is, because the whole argument turns on it. A null result means the trial did not detect a difference between treatment and placebo. It does not mean no difference exists. Three quite different situations produce the same null: the compound does not work; the compound works but was given to patients who did not have the abnormality it corrects; or the compound works in those patients but the trial could not detect the effect, because the dose, endpoint, duration, or sample size was inadequate. Only the first is a statement about the biology. The second and third are statements about the design. A trial that has not characterized the variable it intends to modify cannot distinguish between them, and the conclusion drawn from the antioxidant record—that oxidative stress is not causally relevant—requires the first, while the evidence supports any of the three. Those four are the subjects of Section 4.2, Section 4.3, Section 4.4 and Section 4.5. They share a common element, and it is that element which makes the null results uninformative rather than merely disappointing.

**None of these trials characterized the participant’s redox state before treatment.** Participants were enrolled on clinical diagnosis alone. No compartment was specified. No direction of displacement was established. No regulatory capacity was assessed, whether at rest or under challenge. No target range was defined, and no titration was performed against any redox endpoint. The dose was fixed by protocol rather than by the patient’s position relative to an operating range.

That design is a subtraction: add antioxidant, expect less damage. It is coherent only if oxidative stress is a single quantity, monotonically harmful, with an optimum at zero. Redox biology is none of those three things. The couples are not mutually equilibrated, so there is no single quantity to lower [11]; the relationship to harm is not monotonic, since the signaling range is lost if the variable is driven downward [13]; and the optimum is therefore a position within a range, not a floor. What must be characterized is plural and compartment-specific—the position of particular couples in particular tissues, and the capacity to restore them—which is a more demanding requirement than the damage model imposed and the reason Section 5 dwells on what current biomarkers cannot support.

The field’s own literature had rejected the subtraction model before the trials began. Sies and Cadenas framed the pro-oxidant–antioxidant *balance* of the intact cell or organ as the object of measurement [9]. Sies then stated the definition and its clinical corollary together—oxidative stress as imbalance in that equilibrium, forming the basis of antioxidant therapy—while cautioning that the clinical evidence did not yet permit an unequivocal distinction between causal and associative relationships of pro-oxidants to disease [10]. That was 1991; DATATOP reported two years later [5]. Jones sharpened the model further in 2006, defining oxidative stress as a disruption of redox signaling and control [11]—eight years before QE3 randomized 600 patients to placebo, 1200 mg/day, or 2400 mg/day of coenzyme Q10—doses assigned by protocol rather than titrated to any measured variable—while giving 1200 IU/day of vitamin E to every arm including placebo, without measuring any redox variable in any participant [7].

The homeostatic concept was therefore not overlooked. It was present at the naming of the field and refined throughout the trial era; what did not occur was its translation into trial architecture. The biology described a regulated system; the trials treated a stockpile. That distinction determines what it would take to put things right. If the field had misunderstood the biology, correcting it would require new discovery, and there is no telling how long that takes. But the biology was not misunderstood. It was simply never measured in the patients who were treated. What is missing is not knowledge but an assay—and building an assay is a defined piece of work with a foreseeable end, which is why we regard the problem as solvable rather than merely diagnosed.

Table 2 sets the conceptual record against the trial record. The empty cells are the argument.

**Figure 5:**
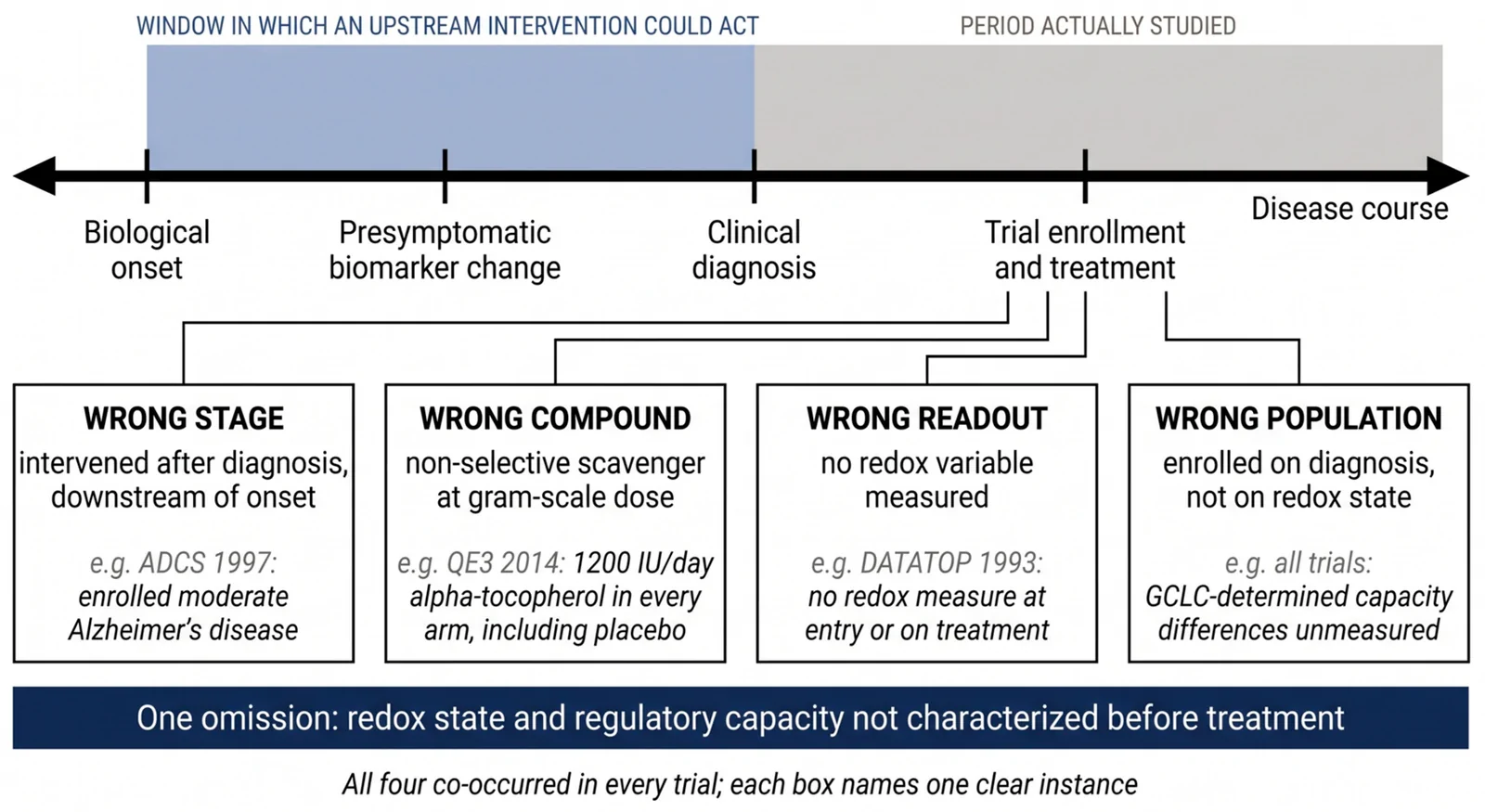
The four failure modes located on the disease course, with one illustrative trial named beneath each. Every trial examined in Section 2 enrolled at or after clinical diagnosis, which falls downstream of biological onset and of the presymptomatic interval in which biomarker change is now detectable [27,28,29]. The named examples are illustrative rather than exclusive: all four failures co-occurred in every trial discussed, and each box identifies one clear instance. The *GCLC* example under WRONG POPULATION is used to name a class of unmeasured constitutional variation rather than to assert that this particular variant is the relevant one in Parkinson’s or Alzheimer’s disease, where it has not been studied. The four are not independent shortcomings but four expressions of a single omission—the individual’s redox state and regulatory capacity were not characterized before treatment, so dose could not be set, titrated, or targeted against the state it was intended to correct. Created in BioRender. Lado, L. https://BioRender.com/t65vjg4 (accessed on 29 August).

The four failures examined below are four expressions of that single omission, appearing at four points in the trial design (Figure 5). **Stage** concerns when the intervention was given relative to the displacement. **Compound** concerns what the drug acted on—a specific couple, or the entire range indiscriminately. **Readout** concerns whether the relevant variable was measured at all. **Population** concerns in whom it was displaced to begin with. Each is separately correctable. None was corrected in any of the trials that defined the field.

### 4.2. Wrong Stage

Every trial discussed in Section 2 intervened after clinical diagnosis. DATATOP and QE3 both enrolled patients described as having *early* Parkinson’s disease, and both defined earliness clinically—by symptom duration and Hoehn and Yahr stage [5,7]. The Alzheimer’s Disease Cooperative Study went further downstream, enrolling patients with moderate disease [6].

The difficulty is that clinical onset and biological onset are not the same event. In Parkinson’s disease, significant dopaminergic neuronal loss has already occurred by the time a clinical diagnosis is made, and subclinical reductions in striatal dopamine transporter binding are detectable in asymptomatic at-risk individuals—Berendse and colleagues identified such reductions in 4 of 25 hyposmic relatives of patients with Parkinson’s disease, two of whom subsequently developed clinical parkinsonism, and in none of 23 normosmic relatives [27].

In Alzheimer’s disease, the point can now be made with quantitative biomarker data: in longitudinal cohorts of individuals who are still cognitively unimpaired, plasma glial fibrillary acidic protein (GFAP), an astrocyte marker, rises with the shift from soluble to insoluble amyloid-β, and cerebrospinal fluid chitinase-3-like protein 1 (YKL-40) tracks the subsequent steps from amyloid to tau pathology and from tau to neuronal injury; in formal mediation analysis, each carries part of the statistical effect of the earlier step on the later one [28]. Microglial activation, indexed by soluble triggering receptor expressed on myeloid cells 2 (sTREM2), is detectable at the stage of subjective cognitive decline, before mild cognitive impairment [29].

If the redox contribution to these disorders is upstream—a driver of the cascade rather than a marker of its later stages—then a trial beginning at diagnosis starts after the period in which the intervention could plausibly act. A null result under those conditions is uninformative about upstream causality. It establishes only that the intervention does not reverse established disease, which was never the strong version of the hypothesis.

### 4.3. Wrong Compound

The compounds tested were, with few exceptions, non-selective stoichiometric scavengers administered at gram-scale or near-gram-scale doses. This is a problem on three counts.

**Selectivity.** Hydrogen peroxide in the low nanomolar range functions in redox sensing and signal transduction; damage predominates only at concentrations above roughly 100 nM [13]. A scavenger that lowers reactive species without regard to species, compartment, or concentration cannot selectively remove the damaging fraction. It compresses the entire range.

**Dose.** The doses used exceed the thresholds at which harm has been demonstrated in other populations. Two findings establish those thresholds.

Miller and colleagues pooled 19 randomized trials with 135,967 participants. Vitamin E at 400 IU/day or above was associated with increased all-cause mortality, with a significant dose–response relationship and risk emerging above 150 IU/day [30]. The pooled risk difference was 39 additional deaths per 10,000 people treated (95% CI 3 to 74; *p* = 0.035), roughly one death for every 256 treated. That is a small individual risk, and it would be an acceptable one against a demonstrated benefit. Against no benefit, it is not a trade at all.

SELECT randomized 35,533 healthy men to 400 IU/day and found increased prostate cancer incidence (HR 1.17; 99% CI 1.004–1.36; *p* = 0.008) [31]—a 17% relative increase in men taking a supplement expected to protect them. Here the direction of effect, not merely the absence of one, is what makes the result informative.

Set those thresholds against the neurology trials. DATATOP, the Alzheimer’s trial, and TEAM-AD each gave 2000 IU/day, and TEAM-AD reported functional benefit at that dose [8]; QE3 gave 1200 IU/day to every arm, including placebo. Two thousand IU/day is five times the 400 IU/day at which harm was demonstrated and thirteen times the 150 IU/day above which risk began to rise; 1200 IU/day is three times and eight times, respectively. Table 3 sets these out, expressed as multiples of 400 IU/day. None of the four trials titrated to a measured variable, because none measured one. The trials were not merely testing a compound that may not have helped; they were administering it indefinitely and without monitoring, at multiples of a threshold at which it had been shown to do harm elsewhere.

We note the comparison rather than assert equivalence. The populations differ, and the neurology trials were not powered to detect mortality. The relevant observation is simply that in all three, dose was set without reference to any measured redox variable—because none was measured. TEAM-AD, which gave the same 2000 IU/day for a mean of 2.27 years, reported no difference in all-cause mortality for alpha-tocopherol [8]; that observation limits how far the pooled mortality signal can be carried into the neurology trials, and we do not carry it farther than the table shows.

**Direction of effect.** Alpha-tocopherol is not uniformly an antioxidant. In lipoproteins it displays neutral, anti-, or pro-oxidant activity depending on conditions through a mechanism termed tocopherol-mediated peroxidation, in which the alpha-tocopheroxyl radical (α-TO•) reacts with polyunsaturated lipids in the absence of an effective co-antioxidant [32,33]. Upston and colleagues advanced precisely this context dependence to rationalize the otherwise confounding null effect of vitamin E on atherosclerosis across intervention studies [32].

The parallel to the neurodegeneration literature is suggestive rather than established: this work concerns low-density lipoprotein, not brain tissue, and the co-antioxidant environment of the central nervous system differs. We note it because it demonstrates that a compound’s direction of effect can invert with context—which is precisely what a dysregulation model predicts and a damage model does not—and because the slight adverse trends in both QE3 active arms [7] are at least consistent with pharmacology rather than noise. We do not claim these trials caused harm.

### 4.4. Wrong Readout

A trial can only detect a redox effect if it measures one. Most did not, relying instead on clinical endpoints alone. Where oxidative markers were measured, their validity was not straightforward.

Malondialdehyde (MDA), most often assayed by thiobarbituric acid reactive substances (TBARS), and the F2-isoprostanes have both served as indices of in vivo lipid peroxidation. The Biomarkers of Oxidative Stress Study examined how robust these measures are to pharmacological interference. The work was conducted in a rat model of carbon tetrachloride poisoning rather than in patients, and we note that limitation, but the finding bears on the interpretation of the same assays in human studies: both isoprostane and MDA formation could be partially inhibited by the cyclooxygenase inhibitors indomethacin and meclofenamic acid, albeit to differing degrees [34]. The authors’ interpretation was that the reduction likely reflected suppression of non-enzymatic lipid peroxidation rather than loss of catalytic generation, and that these products do primarily constitute markers of oxidative stress [34]. The methodological point stands regardless of species: a widely used redox readout is measurably sensitive to a common concomitant medication.

Two further problems compound this. The first is compartment. Peripheral markers in plasma or urine are routinely used as proxies for central nervous system redox state; the quantitative correspondence between the two compartments has not, to our knowledge, been systematically established for any redox couple, and we treat this as an open methodological question rather than a settled one.

The second problem is the one this paper is principally concerned with. A marker of accumulated *damage* is not a marker of redox *signaling* or of redox *control* [11,13,14]. Lipid peroxidation products record what has already happened to a lipid. They do not report where a thiol/disulfide couple sits within its operating range, nor whether that couple is regulated or has escaped regulation. Under a damage model, this distinction is immaterial, because damage is the phenomenon of interest. Under a dysregulation model, it is the entire question—and it means that a trial could measure MDA and F2-isoprostanes conscientiously at every visit and still have measured nothing about the variable it intended to modify.

### 4.5. Wrong Population

The fourth failure is the most consequential and the most easily corrected. None of the trials measured redox status at entry. Participants were enrolled on diagnosis, and every participant received the intervention regardless of whether there was any evidence of redox abnormality to correct.

This assumes that patients with a given diagnosis are homogeneous in redox capacity. They are not. Gysin and colleagues provided convergent genetic and functional evidence in schizophrenia: cultured fibroblasts from patients challenged with oxidative stress showed 26% lower glutamate cysteine ligase activity than controls (*p* = 0.002), correlating with 29% lower expression of the catalytic subunit GCLC (*p* < 0.001). A trinucleotide repeat polymorphism in *GCLC* was associated with schizophrenia across two independent case–control samples, and subjects carrying disease-associated genotypes had lower GCLC protein expression (*p* = 0.017), lower enzyme activity (*p* = 0.037), and lower glutathione content (*p* = 0.004) than those with genotypes more frequently found in controls [20].

The finding is worth stating in set-point terms. What differs between genotypes is not the amount of damage sustained but the capacity to defend a set-point under challenge—the fibroblast difference emerged when cells were oxidatively stressed. This is a difference in regulatory reserve, invisible to any measurement of accumulated damage at rest, and directly relevant to who should be enrolled.

The implication for trial design is direct. If the capacity to synthesize glutathione under oxidative challenge varies by genotype within a diagnostic category, then a trial that ignores that variation dilutes any true effect across a population in which only a subgroup has the deficit that the intervention targets. The result is a null finding that reflects the enrollment strategy rather than the pharmacology.

### 4.6. The Strongest Counterevidence

If the argument advanced so far is correct, a trial that fixed these design failures should do better than one that did not. Two trials fixed several of them. Both failed. That is evidence against the hypothesis advanced here; it is the strongest such evidence in the published record, and we present it as such rather than looking for reasons to set it aside.

**MitoQ in Parkinson’s disease.** Snow and colleagues randomized 128 patients with newly diagnosed, untreated Parkinson’s disease to one of two doses of MitoQ or placebo for 12 months [24]. MitoQ is a form of coenzyme Q10 engineered to concentrate inside mitochondria rather than disperse through the cell: a ubiquinone moiety conjugated to a lipophilic triphenylphosphonium cation, which drives its accumulation across the mitochondrial membrane. This is the compound selectivity that Section 4.3 argues was absent from the earlier trials. The population addressed a second failure: untreated patients at the point of diagnosis, as close to biological onset as a diagnosed cohort permits. Two of the four failure modes—wrong compound and wrong timing—were therefore corrected in advance. The trial showed no difference from placebo on any measure of progression, and the authors wrote that the finding should be taken into account when considering the oxidative stress hypothesis for the pathogenesis of Parkinson’s disease [24].

**Elamipretide in primary mitochondrial myopathy.** MMPOWER-3 is the more demanding test. Elamipretide is a peptide that concentrates in mitochondria and stabilizes the inner membrane. The trial ran 24 weeks against placebo in 218 participants, every one of whom had *genetically confirmed* primary mitochondrial myopathy [25]. That is the point. These patients were selected by the molecular defect itself, not by a clinical syndrome or a biomarker threshold—which is a stronger form of stratification than this paper asks for anywhere. The trial missed both primary endpoints. Patients receiving elamipretide walked 3.2 m *less* in six minutes than those receiving placebo (95% CI −18.7 to 12.3, *p* = 0.69). The effect was not merely absent; the point estimate favored placebo, and the confidence interval excludes any benefit greater than about twelve meters. Total fatigue score showed the same pattern (−0.07; 95% CI −0.10 to 0.26, *p* = 0.37) [25]. The paper carries a Class I evidence statement that elamipretide does not improve these outcomes. An earlier crossover study of 30 participants had also missed its primary endpoint. Its 6-min walk difference of 19.8 m did favor treatment, but the confidence interval crossed zero (95% CI −2.8 to 42.5, *p* = 0.0833), and several patient-reported measures favored elamipretide while the objective endpoint did not [35].

Taken together, these trials establish that compound selectivity and mechanistically defined enrollment are not jointly sufficient. Three responses are available, and we state all three because the first two are partial.

First, the **stage was not corrected in either trial.** MitoQ enrolled at diagnosis, already downstream of substantial nigrostriatal loss (Section 4.2); MMPOWER-3 enrolled established myopathy. This is consistent with the present argument but weakens it, implying the intervention must be tested before patients become identifiable—a requirement no presently enrollable population satisfies.

Second, **genetic confirmation is not a redox measurement.** A confirmed mitochondrial DNA variant establishes that a lesion exists, not that any particular couple is displaced from its operating range, in which compartment, or in which direction. Since the couples are not mutually equilibrated [11], genotype selects patients who share a lesion, not a redox state. MMPOWER-3 therefore performed a different stratification from the one proposed here. That distinction is real, but it is also convenient, and should not be advanced as though it disposed of the result.

Third, it must be granted that **the hypothesis may be narrower than its proponents suppose**—relevant to some disorders and not others, with Parkinson’s disease and primary mitochondrial myopathy on the wrong side of the line. Nothing here excludes this reading. It is the reading a skeptical reviewer should hold, and Section 6 must be assessed against it rather than around it.

### 4.7. What Follows

The four failures set out in Section 4.2, Section 4.3, Section 4.4 and Section 4.5 are not alternatives. They co-occurred in every trial discussed in Section 2. DATATOP intervened after diagnosis with a non-selective compound at 2000 IU/day, without a redox readout, in an unstratified population [5]. So did the others. Section 4.6 shows that correcting two of the four was still not enough.

A trial constructed this way cannot distinguish between two hypotheses: that oxidative stress is not causally relevant, and that oxidative stress is causally relevant but the trial was incapable of demonstrating it. The field adopted the former. The evidence supports neither over the other.

This paper therefore advances a claim that can be disconfirmed. Consider a trial that satisfies four conditions, using *displacement* in the sense defined in Section 3.1: a couple whose ratio has moved outside its normal operating range, in a stated direction:

1. Redox state is characterized at baseline in the individual patient: which couple, in which compartment, is displaced in which direction.
2. Enrollment is restricted to patients in whom such a displacement is actually demonstrated, rather than assumed from the diagnosis.
3. The intervention is directed at that particular displaced couple rather than at reactive species in general.
4. Target engagement is shown—the displacement is corrected by treatment, and this is measured rather than inferred.

If such a trial returns a null result, the hypothesis that redox dysregulation contributes causally to that disorder should be abandoned. Section 6 sets out what each condition would require in practice.

Until that trial is run, the honest position is that the decisive experiment has not been performed. We regard its absence, rather than the presence of null results from experiments not designed to perform it, as the current state of the evidence.

What has changed is that the omission underlying all four failures is now partly remediable. Redox state can be characterized prospectively in individual patients, in specified compartments, before treatment begins. Section 5 examines how far current biomarkers support this and where they do not.

## 5. What Can Be Measured in Patients Today, and What Cannot

Section 4 argued that the trials failed because they did not measure the variable they intended to modify. This section asks whether that variable can now be measured. The answer is a qualified and uneven “yes,” and the qualifications matter more than the affirmation, because a stratified trial built on an inadequate measure repeats the original error in a more expensive form.

We assess four classes of measurement against three requirements: that the measure reports *redox state* rather than accumulated damage, that it reports it in the *central nervous system*, and that it is *stable enough in an individual* to define a baseline and detect change.

### 5.1. Damage Products: They Confirm Damage, Not Dysregulation

The F2-isoprostanes, MDA, 8-hydroxy-2′-deoxyguanosine, and protein carbonyls fail the first requirement by construction, and it is worth being precise about the sense in which they fail. They are valid measurements of oxidative damage; they occupy the fourth position in the causal sequence set out in Section 3.2. What they cannot do is report where a regulatory couple sits within its range, and therefore cannot by themselves distinguish a causal upstream abnormality from a compensatory or epiphenomenal one. Under a damage model, this is the phenomenon of interest; under the model advanced here, it is a downstream consequence whose relationship to the regulated variable is not fixed [11,13,14]. Their pharmacological sensitivity compounds the problem—both isoprostane and MDA formation were partially inhibited by indomethacin and meclofenamic acid [34], so a trial with unbalanced concomitant nonsteroidal anti-inflammatory drug (NSAID) use may register a redox difference that is pharmacological in origin. These measures retain value as safety and pharmacodynamic readouts, but cannot define the population a stratified trial should enroll.

### 5.2. Central Measurement: Glutathione Magnetic Resonance Spectroscopy

Proton magnetic resonance spectroscopy (MRS) can quantify glutathione in defined brain regions in living patients, which addresses the compartment problem directly. This is the single most important methodological development for the argument advanced here, and its current limitations should be stated plainly.

Girgis and colleagues measured glutathione in the dorsal anterior cingulate cortex and glutamate in the dorsal anterior cingulate and medial prefrontal cortex in 19 patients with schizophrenia and 20 control subjects—39 participants in a study the authors describe as a pilot. Measurements were taken at baseline and 60 min after an acute 2400 mg oral dose of N-acetylcysteine [36]. No differences in glutathione or glutamate were found between patients and controls at baseline or following N-acetylcysteine administration, in either region. The authors concluded that future work should measure glutathione in regions with documented glutamatergic abnormality, or use genetic polymorphisms, while controlling for age and medication status, in order to identify patients more likely to respond [36].

This is a negative result, and it should not be minimized. It indicates that a single-region, single-timepoint glutathione measurement did not separate a schizophrenia cohort from controls in a disorder for which the genetic and functional case for impaired glutathione synthesis is comparatively strong [20]. Four interpretations are compatible with it: that regional selection was wrong; that medication status or age obscured a real difference; that the study was too small to detect a difference that exists; or that resting cortical glutathione concentration is simply not where the abnormality resides.

The third of those requires the same discipline we applied to the trial record in Section 4. This was a pilot study, as described by its authors, with 39 participants in total. A null result at that sample size constrains very little, and we do not present it as evidence that no resting difference exists. We cite it for a different reason: the authors’ own recommendation—to measure in regions with documented glutamatergic abnormality, or to select patients by genetic polymorphism, while controlling for medication status and age—is the stratification argument of this paper, reached independently and from the measurement side [36].

**Figure 6:**
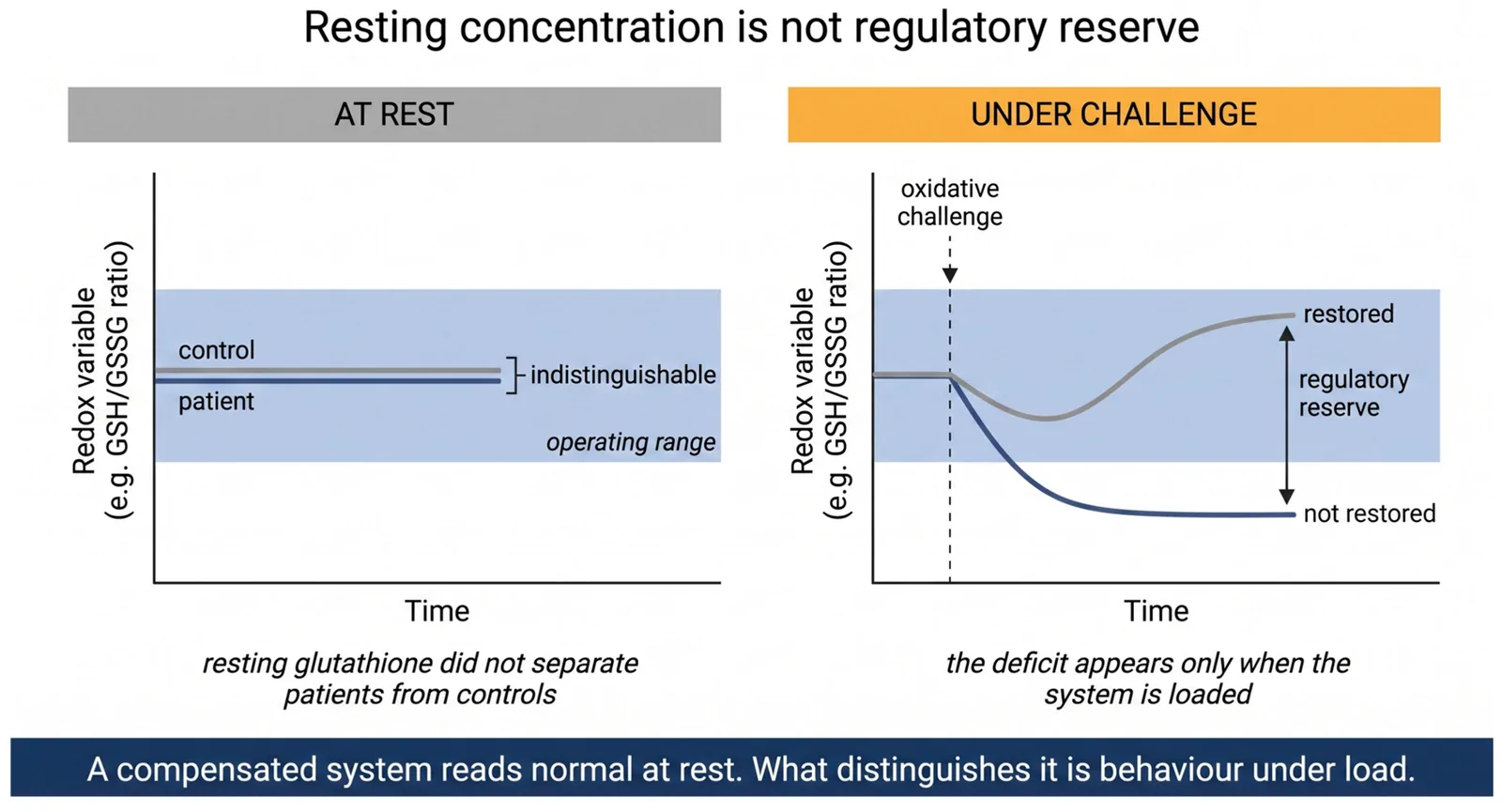
Resting concentration and regulatory reserve are different quantities. (**left panel**, at rest) A compensated system holds its regulated variable within the operating range, and patients are not separable from controls—the pattern reported for cortical glutathione in schizophrenia [36]. The axis is labeled GSH/GSSG as the generic example of a redox couple; we note that the magnetic resonance measurement in that study quantified total glutathione, which does not resolve the oxidized fraction, and the ratio is used here to represent redox position in principle rather than the specific quantity measured. (**right panel**, under challenge) Following an oxidative challenge, both are displaced, but the control system returns the variable to its operating range while the compensated system does not. The vertical separation between the two endpoints is what we term regulatory reserve: the capacity to restore, rather than the resting value itself. The pattern is that reported for glutamate cysteine ligase activity in schizophrenia fibroblasts, where the deficit emerged only under challenge [20]. The curves are schematic and illustrate the distinction; they are not derived from either dataset. Clinically, the left panel is a fasting glucose and the right panel a glucose tolerance test; Section 3.3 develops the comparison. Created in BioRender. Lado, L. https://BioRender.com/wdgwnlc (accessed on 29 August 2026).

The fourth interpretation deserves emphasis because it follows from the argument of Section 4.5. What Gysin and colleagues demonstrated was not a resting deficit but a deficit under oxidative challenge—fibroblast glutamate cysteine ligase activity diverged when cells were stressed [20]. A resting measurement of a regulated variable in a compensated system may be normal precisely because regulation is working. What distinguishes such a system is its behavior when loaded. The two findings are consistent rather than contradictory once the distinction is drawn (Figure 6).

This has a direct methodological consequence, and it qualifies the argument of Section 4 rather than contradicting it: **a static baseline measurement is an improvement over no measurement, but may still be insufficient.** What a dysregulation model requires is redox reserve as defined in Section 3.3—the capacity to restore a compartment-specific variable following a standardized perturbation—rather than resting concentration alone. No such challenge protocol is established for central redox measurement in humans, and its absence is, in our assessment, the principal methodological gap in the field.

### 5.3. Fluid Markers of Neuronal and Glial Injury

Neurofilament light chain (NfL), GFAP, YKL-40, and sTREM2 now permit staging of neurodegenerative processes in living patients with a precision unavailable when the trials of Section 2 were designed [28,29]. Their value to the present argument is real but indirect, and their limits should be stated.

None is a redox measure. They index axonal injury and glial activation, which are consequences of many processes. Neurofilament light chain in particular rises across a wide range of neurological disorders and is not disease-specific; its interpretation depends entirely on the clinical context in which it is measured.

What they can do is address the stage failure of Section 4.2: enrollment after the biology has already advanced. In longitudinal cohorts of cognitively unimpaired people, plasma GFAP, an astrocyte marker, rises with the shift from soluble to insoluble amyloid-β, and cerebrospinal fluid YKL-40 tracks the subsequent steps from amyloid to tau pathology, and from tau to neuronal injury; in formal mediation analysis, each carries part of the statistical effect of the earlier step on the later one [28]. Soluble TREM2, a marker of microglial activation, is already elevated at the stage of subjective cognitive decline before mild cognitive impairment [29]. These markers therefore permit enrollment of patients who are biologically affected and clinically presymptomatic—the population Section 4.2 argues the trials never reached.

Their role in a redox trial is thus to define *when*, not *whom*. That is one of the two things the trials of Section 2 lacked, and it is now available.

### 5.4. Genotype

Redox-relevant polymorphisms—most prominently the *GCLC* trinucleotide repeat associated with schizophrenia across two independent case–control samples and with lower GCLC expression, lower enzyme activity, and lower glutathione content [20]—offer a stable, inexpensive, and readily assayed stratification variable.

Two constraints apply, and the second is the more serious. First, redox-related candidate genes do not reach genome-wide significance in current large-scale association studies of the relevant disorders; these remain candidate-gene findings and must be described as such rather than as diagnostic or screening tools. Second, as MMPOWER-3 demonstrated (Section 4.6), genotype-based stratification is not redox-state stratification [25]. A genotype identifies patients who share a constraint on synthetic capacity. Whether that constraint is currently displacing any particular couple, in any particular compartment, is a separate question that the genotype does not answer.

Genotype is therefore best understood as an enrichment variable rather than a stratification variable—a means of raising the prior probability that a redox abnormality is present, not a demonstration that it is.

### 5.5. Where This Leaves Us: Position Partly Visible, Capacity Not Visible at All

Table 4 sets the four classes against three requirements.

No single class satisfies all three requirements, and the pattern of what is missing is specific rather than general.

**Where the system currently sits can be partly seen.** Glutathione MRS is the only method that reports a regulatory variable in the correct compartment of a living brain. Two limitations attach to it: it quantifies total glutathione and cannot separate the oxidized fraction, and a resting value may read normal in a system that is compensating [36].

**What capacity the system has left cannot be seen at all.** There is no assay, no protocol, and no published attempt in the central nervous system. This is the gap, and it is the one the argument turns on.

**What is measured best is furthest from the mechanism.** Injury markers are validated, sensitive, and available—and they report accumulated damage, which Section 3.2 places at the end of the causal sequence rather than near its origin. Genotype reports a stable constraint but not the current state; damage products report neither.

The measurement failure identified in Section 4.4 is therefore *partly* remediable now: enough to design a better trial than those of Section 2, not enough to design the trial the argument ideally requires. Section 6 sets out what can be built with what exists, and names the gaps that would have to be closed before the hypothesis could be regarded as properly tested.

## 6. Staging: What Neurology Borrowed from Oncology, and What It Did Not

### 6.1. The Argument from Oncology

Oncology does not enroll patients by diagnosis. It enrolls them on a stage, defined by measured extent of a biological process—tumor size, nodal involvement, distant spread—rather than by symptom severity. Prognosis is stated by stage, treatment selected by stage, trials powered within stage. A trial enrolling everyone with a given tumor type regardless of stage would be uninterpretable, for exactly the reason given in Section 4.5.

McGorry and colleagues made this argument for psychiatry two decades ago, observing that clinical staging is widely used elsewhere in medicine and had been largely ignored in psychiatric practice, and proposing it as a means of choosing earlier and safer interventions, of evaluating treatments by their capacity to prevent progression between stages, and of organizing biological findings into a coherent clinicopathological framework [37]. The parallel argument in neurology arrived with the NIA-AA Research Framework, which defines Alzheimer’s disease by underlying pathologic processes documented by biomarkers rather than by its clinical consequences—shifting the definition in living patients from a syndromal to a biological construct and grouping biomarkers into amyloid, tau, and neurodegeneration categories under the AT(N) system [38].

Against that background, the trials of Section 2 are anachronistic in a specific and instructive way. DATATOP and QE3 enrolled patients with early Parkinson’s disease, where earliness was defined by symptom duration and Hoehn and Yahr stage—that is, by the severity of the clinical syndrome [5,7]. The Alzheimer’s trial enrolled patients with moderate disease, likewise a syndromal descriptor [6]. None of these is a stage in the oncological sense. They record how ill the patient appears, not how far a measured biological process has advanced.

This is the same failure identified in Section 4.2, restated in a constructive way. The trials did not merely intervene late; they had no instrument capable of telling them how late.

### 6.2. Why the Analogy Is Imperfect, and What That Requires

The oncological analogy should not be pressed further than it can bear, and two disanalogies are decisive for what follows.

**Tumor burden is scalar; redox state is not.** TNM staging works because tumor burden is a single quantity, measurable in principle by direct observation, and monotonically related to prognosis—more is worse. Redox state possesses none of these properties. The major thiol/disulfide couples are not in redox equilibrium with one another, so there is no single quantity to stage [11]. Worse, the relationship to harm is not monotonic: hydrogen peroxide in the low nanomolar range performs signaling functions that are lost if it is driven downward, and damage predominates only above roughly 100 nM [13]. A staging system that treated redox burden as a scalar to be minimized would reproduce, in more sophisticated notation, the subtraction model that Section 4.1 identifies as the original error.

**Tumor stage is a state; redox displacement may be a rate.** This follows from the pattern in Section 4.6 and from the two successful trials. Edaravone succeeded when enrollment required a documented decline of one to four points on the Revised ALS Functional Rating Scale (ALSFRS-R) during a twelve-week run-in period—that is, when patients were selected on a *measured rate of change* in an active process, not on a static classification [15]. The point must be stated precisely: edaravone’s enrichment criteria were clinical and functional, not redox-specific. It provides proof of principle for process-based enrichment, not proof of redox-based enrichment, and we advance it only in the former sense. MMPOWER-3, by contrast, stratified by genotype: a stable, lifelong property that establishes that a lesion exists but says nothing about whether it is currently active [25]. The trial that selected on process activity succeeded; the trial that selected on a fixed molecular characteristic did not.

A redox staging framework must therefore be plural rather than scalar, must specify direction rather than only magnitude, and must incorporate rate as well as state. These are demanding requirements, and no existing instrument satisfies them.

### 6.3. A Proposed Framework

**Figure 7:**
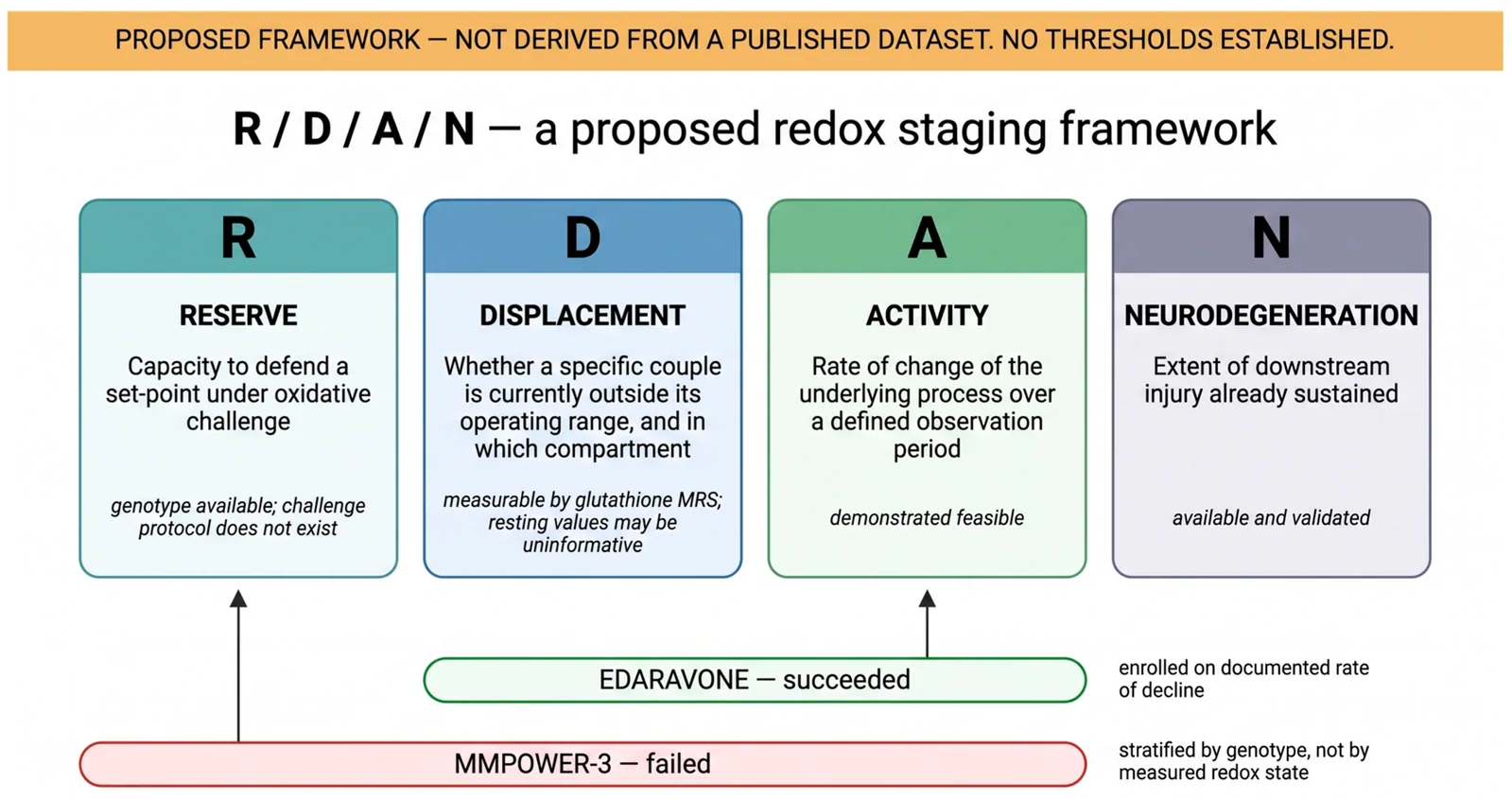
A proposed four-axis redox staging framework. The axes separate regulatory capacity (R), current displacement of a specific redox couple (D)—a paired reduced and oxidized species, such as reduced and oxidized glutathione (GSH/GSSG), whose ratio sets the local redox state—rate of change in the underlying process (A), and accumulated downstream injury (N), because the trial record indicates these behave differently: edaravone selected on measured rate of decline and succeeded [15]—an enrichment criterion that was clinical rather than redox-specific, and therefore evidence for process-based enrollment rather than for redox stratification—whereas MMPOWER-3 stratified by genotype, a stable property closest to the reserve (R) axis, and returned a null result [25]. **The framework is proposed rather than validated. It is not derived from any published dataset, no cut-points are specified because none have been established, and it requires prospective validation in a single cohort before use.** Created in BioRender. Lado, L. https://BioRender.com/y83328g (accessed on 29 August 2026).

Table 5 sets out a candidate structure, shown schematically in Figure 7. **It is proposed rather than validated: it is not derived from any published dataset, and no cut-points are specified because none have been established, and it is offered as a hypothesis about the form such an instrument should take rather than as an instrument ready for use.**

The structure deliberately separates capacity (R), current state (D), rate (A), and accumulated consequence (N). Section 4.6 showed why: MMPOWER-3, stratified by genotype, is the measure closest to R and obtained a null result [25]; edaravone was selected on rate of decline, the A axis, and succeeded [15]. Collapsing these into a single index would lose precisely the distinction that the trial record supports. Section 1 listed five clinical facts; they map onto four axes because two of them, accumulated oxidative damage and irreversible neuronal injury, are both consequences already sustained, neither reports the state of the regulator, and both are therefore recorded on N. Three limitations attach to the framework as a whole. No cut-points are specified on any axis, because none have been established. No challenge protocol exists, so the R axis cannot currently be measured except through the genotype proxy of Section 5.4. And the evidence that the A axis separates responders from non-responders rests on a single trial, edaravone, whose enrichment criterion was a clinical rate of decline and not any redox variable [15].

We note explicitly that the accumulated-injury (N) axis is the only one currently supported by validated instruments, and that it is the axis furthest from the mechanism this paper argues is causal. That is an uncomfortable position, and we do not disguise it.

### 6.4. What a Trial Built on This Would Require

Four elements follow, in descending order of current feasibility.

1. **Enroll on measured process activity, not on diagnosis.** A run-in period establishing rate of change, on the edaravone model [15]. This is demonstrated, requires no new technology, and is the single change most strongly supported by the trial record.
2. **Stage on downstream injury markers to establish position on the disease course.** NfL, GFAP, YKL-40, and sTREM2 identify patients who are biologically affected but still clinically presymptomatic [28,29]. Like element 1, this requires no new technology.
3. **Measure the target couple in the target compartment at baseline and titrate to it.** Regional glutathione MRS is the only current candidate [36]. It is feasible but of unproven informativeness at rest.
4. **Develop a redox challenge protocol.** The measure a dysregulation model actually requires is regulatory reserve under load, not resting concentration—as the fibroblast findings under oxidative challenge indicate [20] and the negative resting MRS result suggests [36]. Nothing of this kind has been established in humans. This is the rate-limiting gap.

Enrollment on measured process activity and staging on injury markers, the first two elements, are enough to build a trial materially better than any discussed in Section 2. The other two, measuring the target couple at baseline and a validated challenge protocol, are required before a null result could count as disconfirming the hypothesis rather than merely failing to confirm it; that is the standard that the falsification criterion in Section 4.7 sets. Until those two exist, a trial built on enrollment and staging alone tests process-based enrollment. It does not test the redox hypothesis.

### 6.5. The Prognostic Argument

A staging system’s value is not confined to trial design, and the clinical case may be the stronger one. Staging supplies prognosis: it lets a clinician tell a patient not only what they have but where they are within it, and allows treatment intensity to be matched to position on the course—the rationale McGorry and colleagues advanced for psychiatry [37], and the reason the NIA-AA framework was built for research use pending validation [38].

Current practice entirely lacks this for redox pathology. There is no equivalent of a stage II designation indicating that a redox-relevant process is active, at a defined point, with a defined expected trajectory. That absence explains why redox findings have accumulated for four decades without changing management: a finding that cannot be located on a course cannot guide a decision about timing, and timing is what an upstream intervention consists of.

Four consequences follow if such a framework could be validated, and they are worth stating explicitly because they are what the proposal is about.

**It separates patients who share a diagnosis but not a lesion.** Two patients with the same clinical diagnosis may differ on R, on D, or on both. Under current practice, they enter the same trial arm and receive the same compound at the same dose. Section 3.3 sets out three molecularly distinct failure modes, and Table 1 sets out the intervention classes they imply; without an instrument that distinguishes them, any true effect in one subgroup is diluted across patients for whom the compound is mechanistically inappropriate.

**It makes timing a variable rather than an accident.** The activity (A) axis records whether the process is currently active and at what rate. Enrollment on diagnosis alone captures patients at unknown and heterogeneous points on their course. Edaravone succeeded when a run-in established the rate of decline before randomization [15], which is the activity (A) axis in operation, even though its criteria were clinical rather than redox-specific.

**It permits target engagement to be demonstrated rather than assumed.** If D is measured at baseline and again on treatment, a null result becomes interpretable: either the displacement was corrected, and the disorder progressed regardless, which disconfirms the hypothesis for that population, or it was not corrected, which indicts the compound rather than the mechanism. Every trial in Section 2 lacked this, and that is why their null results carry so little information.

**It supplies prognosis where none currently exists.** R and N together describe capacity and accumulated injury. Two patients at the same clinical stage but differing in reserve are not in the same position, and a framework that records the difference can inform how intensively to intervene and how early.

We do not claim that such a framework would by itself improve outcomes, and we note that its four axes differ markedly in measurement maturity: accumulated injury (N) is supported by validated instruments and is furthest from the proposed mechanism, while reserve (R) depends on a challenge protocol that does not yet exist. We claim only that in its absence the causal question posed in Section 1 cannot be answered, because the experiment that would answer it cannot be designed.

## 7. Early Detection and the Obstacles to Using It

### 7.1. A Clarification About What Is Being Detected Early

The phrase *early detection of damage* requires care in the context of this hypothesis, because the argument of Section 4 and Section 5 is that damage is the wrong target. Detecting damage earlier is still detecting damage; it identifies a process that has already produced an irreversible consequence, only sooner. If oxidative dysregulation is upstream, the quantity worth detecting early is the displacement of a regulated variable, and damage products are its trailing indicator.

The distinction is practical rather than pedantic. A screening program built on F2-isoprostanes or 8-hydroxy-2′-deoxyguanosine would identify people in whom oxidation has already occurred—a population defined by consequence. A program built on the reserve (R) and displacement (D) axes of Table 5 would identify people whose regulatory capacity is constrained or whose set-point is displaced, before consequence accrues. Only the second is preventive in the sense intended here.

What current biomarkers permit is intermediate, and it is worth stating precisely what they can and cannot do.

### 7.2. What Presymptomatic Detection Currently Achieves

Three capabilities have been established and are available now.

Identification of biologically affected, clinically normal individuals. In cognitively unimpaired people, plasma GFAP rises with the shift from soluble to insoluble amyloid-β, and CSF YKL-40 tracks the subsequent steps toward tau pathology and neuronal injury, each carrying part of the statistical effect of the earlier step in longitudinal mediation analysis [28]; soluble TREM2 is elevated at subjective cognitive decline [29]; reduced striatal dopamine transporter binding is detectable in asymptomatic hyposmic relatives, some of whom later developed clinical parkinsonism [27]. The presymptomatic window is measurable, not hypothetical.

**Enrollment at scale based on biomarker criteria.** The A4 Study enrolled 1147 cognitively unimpaired older adults selected by elevated amyloid on florbetapir PET, across 67 sites in four countries, and followed them to 240 weeks [39]. The trial did not demonstrate a treatment benefit with solanezumab, but it settled a separate question decisively: biomarker-defined presymptomatic enrollment is operationally feasible at multicenter scale. The infrastructure objection to preventive trial design no longer holds.

**Detection of subtle decline within that window.** In A4, both participant- and study-partner-reported cognitive function worsened over the study period, and the correlation between study-partner report and objective performance strengthened over time, most markedly in the highest amyloid tertile [39]. Change is detectable in people who do not yet meet diagnostic criteria—which is what the activity (A) axis of Table 5 requires.

**Figure 8:**
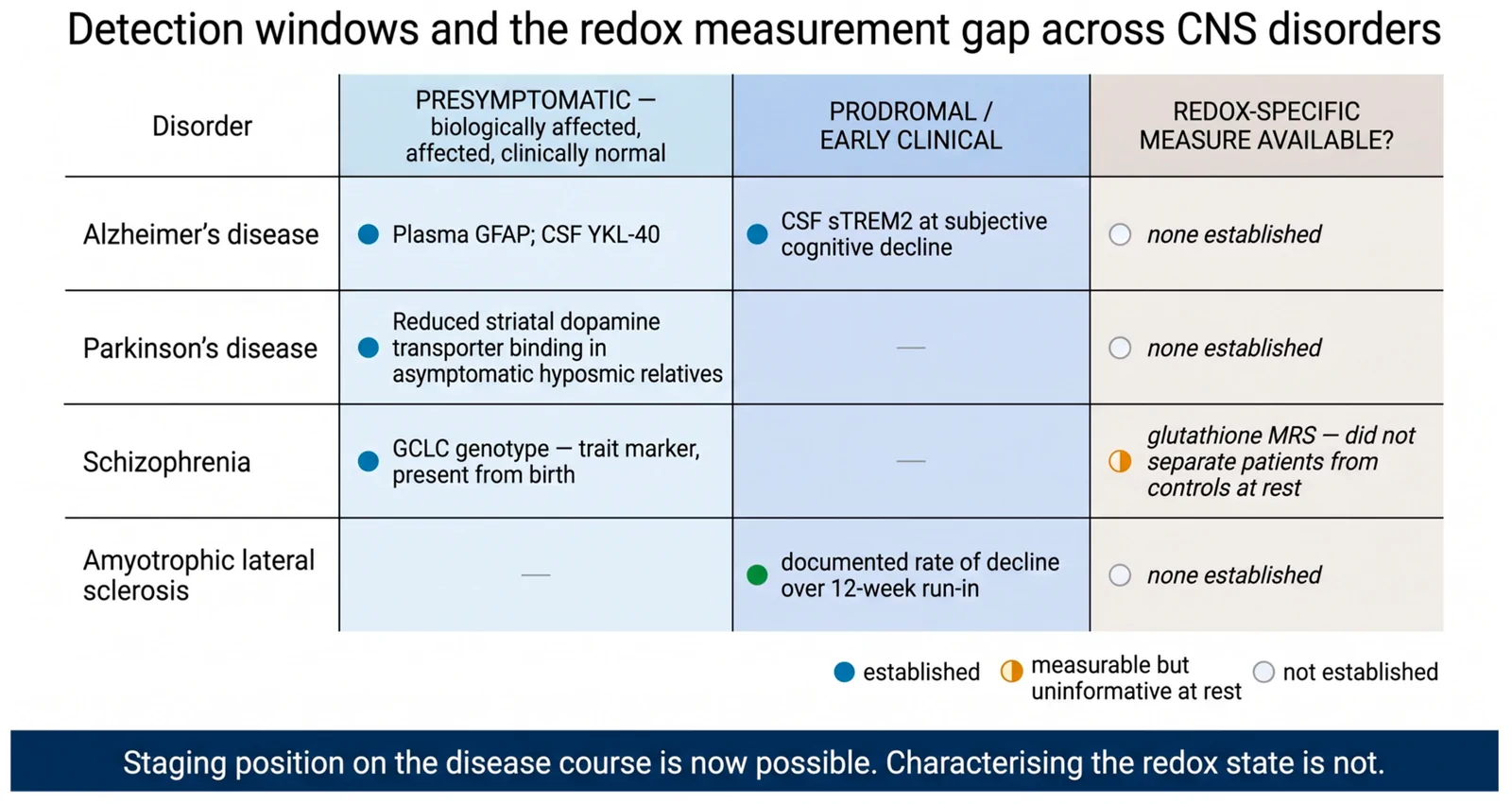
Detection windows and the redox measurement gap across central nervous system disorders. Markers permitting identification of biologically affected but clinically normal individuals are established in Alzheimer’s disease [28,29], Parkinson’s disease [27], and, as a trait marker, schizophrenia [20]; enrollment on documented rate of decline is established in amyotrophic lateral sclerosis [15]. No disorder has an established redox-specific measure. Glutathione MRS is measurable in schizophrenia but did not distinguish patients from controls at rest [36], consistent with a regulatory deficit that appears under challenge rather than at baseline [20]. Cells marked with a dash indicate that no marker meeting the column definition is established in the evidence reviewed here. The green marker in the amyotrophic lateral sclerosis row denotes an established marker and is equivalent to blue; the color distinguishes the prodromal column only. Created in BioRender. Lado, L. https://BioRender.com/vwjdvn5 (accessed on 29 August 2026).

Taken together, these establish elements 1 and 2 of Section 6.4 (Figure 8). What they do not establish is any redox-specific capability: none of these markers report redox state, and a trial using them alone would stage position on the disease course without characterizing the mechanism it proposed to modify.

### 7.3. Methodological Obstacles

**No challenge protocol exists.** This remains the rate-limiting gap identified in Section 5.2 and Section 5.5. Resting glutathione by MRS did not distinguish patients from controls in schizophrenia [36], whereas the genetic and functional deficit in that disorder appears under oxidative challenge [20]. Until a safe, standardized, reproducible redox challenge is developed for human use, the reserve (R) axis cannot be measured directly, and the displacement (D) axis may be measured at the wrong moment.

**Compartment correspondence is unresolved.** Peripheral markers continue to be used as proxies for central redox state without an established quantitative correspondence. MRS addresses this for glutathione in defined regions but not for other couples.

**Long horizons and small effects.** A preventive trial in a presymptomatic population requires years of follow-up, as A4’s 240-week design illustrates [39], with correspondingly large sample sizes and attrition.

### 7.4. Regulatory and Economic Obstacles

**Endpoints.** Regulatory acceptance of biomarker endpoints in presymptomatic populations remains limited; the NIA-AA framework was designated for research use, with its authors cautioning against premature application in practice [38]. A redox staging framework would face the same constraint at a far earlier stage of validation.

**Compound economics.** The compounds most implicated are off-patent—tocopherol, N-acetylcysteine, coenzyme Q10—and cannot support a multi-year preventive trial with biomarker screening. This is not incidental to the historical record: the trials that were run were the trials that someone could afford. Edaravone and omaveloxolone both reached approval as proprietary agents in defined populations [15,16], which is the economic model that worked.

**Screeningcosts.** A redox trial requiring MRS, genotyping, and a run-in period compounds the screening burden already evident in A4 [39]. Enrichment by genotype (Section 5.4) is the most plausible route to a tractable ratio.

None of these obstacles is a scientific objection to the hypothesis. They are reasons the decisive experiment has not been performed, which is a different matter and one worth distinguishing clearly from evidence that it would fail.

### 7.5. Broader Implications and Testable Predictions

The argument advanced here has been made for central nervous system diseases. The chemistry it rests on is not neural, and the boundary deserves examination rather than assumption.

Lipid peroxidation is autocatalytic. A radical abstracts hydrogen from a polyunsaturated fatty acid; the resulting carbon-centered radical reacts with molecular oxygen; the peroxyl radical abstracts hydrogen from a neighboring lipid, regenerating the initiating species. One initiation event can consume many substrate molecules before termination. This is a self-propagating chain, and its rate is governed not by how much oxidant is present but by the balance between propagation and termination.

**Figure 9:**
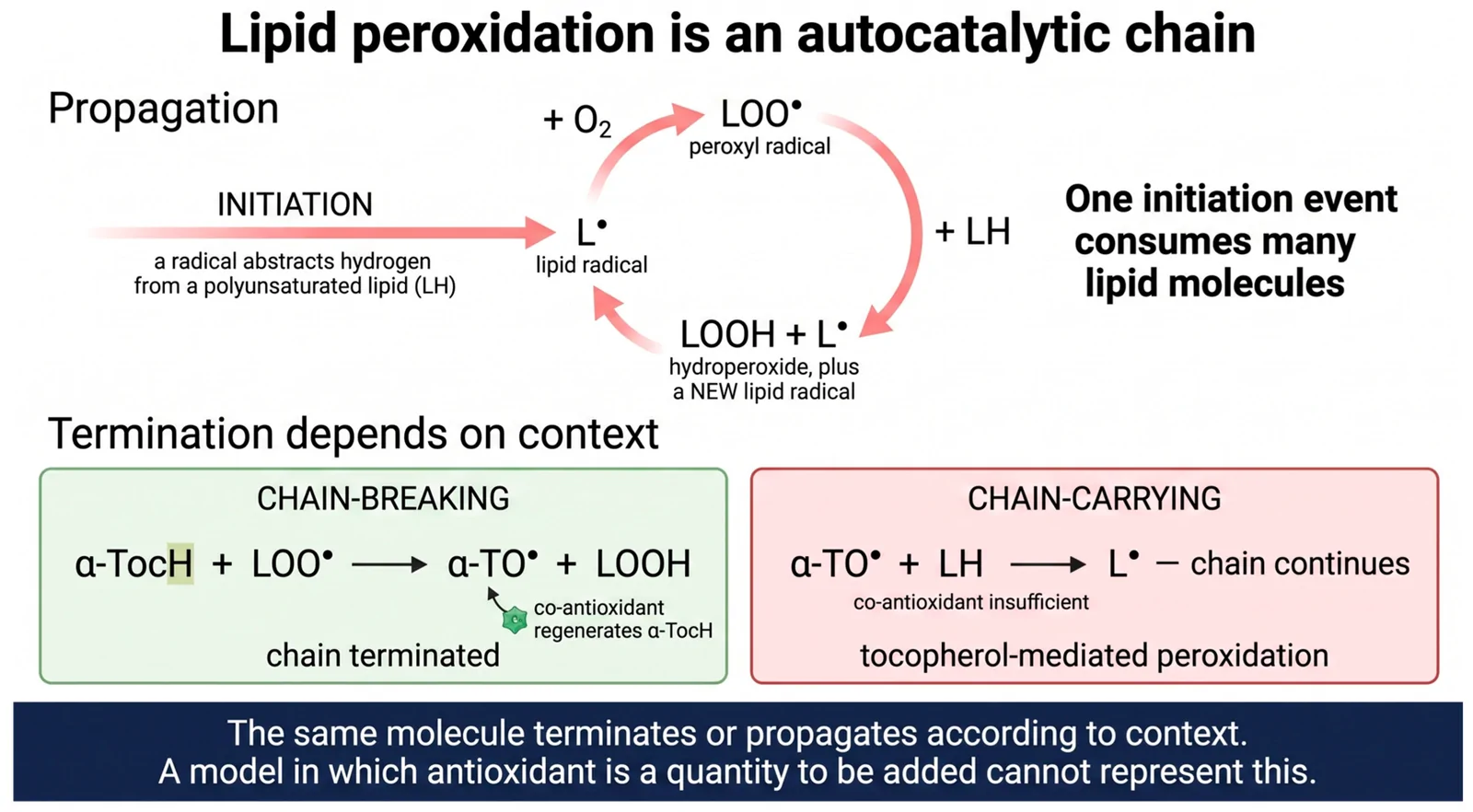
Lipid peroxidation as an autocatalytic chain, and the context dependence of its termination. Initiation abstracts hydrogen from a polyunsaturated lipid; the resulting carbon-centered radical reacts with molecular oxygen; the peroxyl radical abstracts hydrogen from a neighboring lipid, yielding a hydroperoxide and regenerating the initiating species, so that a single initiation event can consume many substrate molecules. Termination is not a property of the antioxidant alone. α-Tocopherol reacting with a peroxyl radical yields the α-tocopheroxyl radical, which is reduced back to α-tocopherol, where an effective co-antioxidant is available, terminating the chain; where co-antioxidant is insufficient, the same radical abstracts hydrogen from a polyunsaturated lipid and propagates it, regenerating α-tocopherol in the process (α-TO• + LH → α-TocH + L•; the panel shows only the lipid product) [32,33]. Neither the chemistry nor its context dependence is specific to neural tissue. Created in BioRender. Lado, L. https://BioRender.com/pi1bli6 (accessed on 29 August 2026).

That framing sharpens the compound argument of Section 4.3. α-Tocopherol is conventionally chain-breaking, but under conditions of insufficient co-antioxidant, the α-tocopheroxyl radical (α-TO•) reacts with polyunsaturated lipids and *carries* the chain rather than terminating it—tocopherol-mediated peroxidation [32,33]. The same molecule is chain-breaking or chain-propagating according to context (Figure 9). A model in which an antioxidant is a quantity to be added cannot represent this. A model in which the system has an operating range, and in which position within that range determines the direction of effect, represents it exactly.

Nothing in that chemistry is specific to neurons. Neither is the evidence already examined here, nor the conclusion others have drawn from it: the same translational diagnosis has been reached independently for the lung, where indiscriminate antioxidant suppression is likewise held to have ignored the signaling roles and spatiotemporal compartmentalization of reactive species [17]. Two organ systems, two literatures, one failure signature. Miller and colleagues pooled trials in general and mixed-disease populations and found excess all-cause mortality above 400 IU/day [30]. SELECT found an increased prostate cancer incidence at that same dose [31]. Upston and colleagues advanced context-dependent tocopherol behavior specifically to rationalize the null effect of vitamin E on atherosclerosis [32]. Three organ systems outside the nervous system, and the same signature in each: a compound that behaves differently by context, given at a fixed dose to an unstratified population, without measurement of the variable it was intended to modify.

This raises a question the present paper can pose but not answer. If redox dysregulation is an upstream process rather than a tissue-specific one, then disorders that co-occur more often than chance should be examined for a shared upstream lesion rather than treated as unrelated conditions in the same patient.

Schizophrenia and type 2 diabetes are the most examined instances. The conventional explanation is antipsychotic exposure, and that explanation is partly correct but not complete. Garcia-Rizo and colleagues compared 84 antipsychotic-naïve patients with a first episode of non-affective psychosis against 98 matched controls: the prevalence of metabolic syndrome did not differ (6% versus 4%, *p* = 0.562), but abnormal glucose homeostasis did (14% versus 5%, *p* = 0.034) [40].

We state plainly what that does and does not establish. It does not demonstrate that oxidative stress mediates the association, and we do not claim it does; the evidence for a shared redox mechanism in this comorbidity is at present thin. What it establishes is narrower and sufficient for the argument: a metabolic abnormality is detectable before pharmacological treatment begins, so the association cannot be attributed wholly to drug exposure. That is the precondition for asking whether a shared upstream process exists—a question that has largely not been asked because the two disorders belong to different specialties.

The systemic hypothesis can be stated in falsifiable form. If redox dysregulation is a shared upstream process rather than a tissue-specific epiphenomenon, then three predictions follow. First, regulatory reserve measured in one accessible compartment should carry predictive information about risk in an anatomically distant one. Second, individuals with constitutionally constrained reserve—the *GCLC* genotype of Section 3.3 provides a candidate—should show excess incidence distributed across organ systems rather than concentrated in one. Third, an intervention that restores the endogenous regulator rather than supplementing its output should shift more than one outcome in the same patient.

Each prediction is testable in existing cohorts with existing assays. To our knowledge, none has been tested directly. We note, as elsewhere in this paper, that the absence of the experiment is not evidence of its result.

Two cautions bound this section. The literature on oxidative stress outside the nervous system is very large, and we have not surveyed it; the claim advanced here is structural rather than comprehensive, and is offered as a direction for investigation rather than a conclusion drawn from one. The co-occurrence of two disorders admits many explanations—shared genetic architecture, shared environmental exposure, ascertainment, and reverse causation among them—of which a shared redox lesion is one candidate and not the most established.

## 8. Conclusions

The therapeutic record of antioxidant intervention in neurological and psychiatric disease is close to empty, with one functional exception [8], and the field concluded from this that oxidative stress is a consequence of neurodegeneration rather than a driver of it. This paper has argued that the conclusion outran the evidence.

The concept of oxidative stress was homeostatic from the outset—a disturbance of a regulated balance [9,10], later refined into a disruption of redox signaling and control across couples that are not mutually equilibrated [11]. However, it was investigated as accumulated damage: measured after the fact, where it was measured at all, and treated by subtraction. Every major trial administered a fixed dose of a non-selective compound to patients enrolled on clinical diagnosis, without establishing any baseline redox variable or titrating to any redox endpoint. Four failure modes followed from that single omission, and all four co-occurred.

A mechanistic reading reinforces the procedural one. The body possesses an inducible antioxidant system under transcriptional control, and, on the reading advanced here, that system fails in at least three distinct ways—a sensor that does not engage, a response that engages but is overwhelmed, and an effector arm with a genetically constrained ceiling [19,20]. None is repaired by supplying antioxidants from outside. Every supplementation trial reviewed here returned a null result except TEAM-AD, whose functional benefit was obtained at a fixed dose without redox measurement [8]; the one positive result that acted on the endogenous regulator instead was omaveloxolone, in a phase 2 trial of 103 patients with a 2.4-point mFARS difference at 48 weeks [16,18]. That is a narrow evidential base, and we do not present it as more, but it points in the same direction as the argument.

We have not claimed this exonerates the hypothesis. Two trials corrected compound selectivity and, in one case, stratified by molecular mechanism, and both returned null results [24,25]; the second carries a Class I statement of no benefit. That evidence is real, and it constrains what may be claimed. The most economical reading of those two results is that the hypothesis is narrower than its general statement, that it applies in some disorders and not in others, and that Parkinson’s disease and primary mitochondrial myopathy may lie outside its scope. Nothing in this paper excludes that reading, and a reader who holds it is reading the trial record correctly. The framework that follows should be judged as a proposal for the disorders in which the hypothesis survives, not as a claim that it survives everywhere. What distinguished the two trials that succeeded was neither compound class nor genotype but the enrollment criterion: selection on a documented, active process [15] or on a defined pathway lesion in a monogenic disorder [16]. Neither criterion was redox-specific, and we therefore advance them as proof of principle for process- and mechanism-based enrollment rather than as validation of redox stratification, which remains untested.

The instrument the field lacks is a staging framework. Oncology enrolls, treats, and prognosticates by measured extent of a biological process; psychiatry and neurology have adopted this only partially [37,38], and not at all for redox pathology. The framework proposed in Table 5 separates regulatory reserve, current displacement, process activity, and accumulated injury, because the trial record indicates these behave differently. It is a structural hypothesis, not a validated instrument, and it requires prospective single-cohort validation before it could be used. It specifies no cut-points, its reserve axis has no assay, and its activity axis rests on one trial whose enrichment criterion was clinical rather than redox-specific [15].

Two of its four axes—current displacement of a specific couple, and accumulated injury—can be measured now, and presymptomatic biomarker-defined enrollment has been shown feasible at multicenter scale [39]. The rate-limiting gap is the absence of any established redox challenge protocol in humans—the measure a dysregulation model actually requires, since a regulated variable in a compensated system may read normal at rest precisely because regulation is working.

One broader implication follows, and we state it as an implication rather than a finding. It is strengthened by the observation that the same translational diagnosis has been reached independently for pulmonary disease [17]. The chemistry underlying this argument is autocatalytic and not tissue-specific, and the trial evidence examined here spans the nervous system, the vasculature, and the prostate with the same signature in each [30,31,32,33]. If redox dysregulation is genuinely upstream, then investigating it organ by organ, within specialty boundaries, may itself be part of why four decades of findings have not converged. Section 7.5 sets out three predictions by which that proposition could be tested or discarded.

The immediate next step is not a trial. It is a methods study. Of the four axes of Section 6.3—reserve, displacement, activity, and accumulated injury—reserve is the one on which the argument turns and the only one with no instrument at all: there is no validated protocol for measuring redox reserve in a living human, and until there is, patients cannot be enrolled on the variable the hypothesis concerns. Developing and validating such a protocol—a standardized perturbation, a defined recovery window, a measurable compartment-specific endpoint—is therefore prior to any therapeutic trial built on this framework and is a smaller and more tractable undertaking than the trials it would make possible. Section 5.2 sets out why a resting measurement will not substitute, and Section 6.3 what the axis would have to deliver.

It is worth stating plainly what would change for patients if this argument is correct, because a paper of this kind can otherwise read as an argument about method alone. Three things follow, and they differ in how much new work each requires.

The first requires none. Patients with these disorders are still advised, and still advise themselves, to take high-dose antioxidant supplements. The evidence assembled in Section 4.3 indicates that at the doses used in the neurological trials this is not a neutral act: excess mortality was detectable above 150 IU/day, and the trials administered eight to thirteen times that amount. If the compounds do not work by the mechanism assumed, the risk is being carried for nothing. That conclusion follows from the existing evidence and needs no further study.

The second requires reanalysis rather than discovery. If a compound can fail in an unselected population and succeed in a selected one, as edaravone did [15], then the trial record does not identify which compounds are inert—only which were given to unselected patients. Some of what has been tested and set aside may be effective in the right patients. Establishing that is a matter of enrollment criteria, not of new chemistry, and it is considerably cheaper than developing new agents.

The third is the substantial one. Three distinct lesions in the endogenous system imply three distinct classes of intervention, and a patient given a compound matched to the wrong lesion is not undertreated but mistreated. Acting on that requires the measurement capability this paper argues does not yet exist. It is the reason that the immediate next step is a methods study rather than a trial, and the reason that we place the challenge protocol before anything therapeutic.

None of this constitutes a treatment, and we do not present it as one. What it constitutes is a claim that a question with real clinical consequences has been closed prematurely, and a specification of what would have to be done to open it properly.

Our claim therefore remains the narrow one stated at the outset. We do not argue that oxidative dysregulation is the primary cause of any neurodegenerative or psychiatric disorder. We argue that the question remains open, that it is open because the decisive experiment has not been designed rather than because it has been performed and failed, and that the conditions under which the hypothesis should be abandoned can now be specified. Should a trial enroll on measured process activity, stage position on the disease course, characterize the displaced couple in the relevant compartment at baseline, direct an intervention at that couple, and still show no effect, then the hypothesis should be set aside. That trial has not been run.

## Figures and Tables

**Table 1:** Three modes of failure in the inducible antioxidant system. The system has three parts in series—a sensor (KEAP1), a transcriptional regulator (NRF2), and an effector arm (glutamate cysteine ligase and the thioredoxin and peroxiredoxin systems)—and each mode below is a failure at a different point along it: the sensor-to-regulator step in the first, the overall output of the system in the second, and the effector arm alone in the third. The distinction is therapeutic rather than taxonomic: the three imply different interventions, and no measurement of accumulated damage distinguishes between them. The final column states the class of intervention that follows from each lesion, developed in Section 3.4. These are mechanistic matches, not established indications; none has been tested against a redox-characterized population, and the compounds named are cited for their mechanism of action rather than as evidence of efficacy in the disorder listed. The three modes are mechanistic interpretations of the findings cited in the “Reported in” column, not established classifications of the disorders named; each rests on a single study, and none has been confirmed in an independent cohort.

Where the System Fails	What Is Broken There	What Is Seen Under Challenge	Reported in	What Treatment Would Follow
**1. Sensing and translocation failure**—at the sensor-to-regulator step	NRF2 stays in the cytoplasm; transcription is not induced despite oxidative stress being present	No response mounted	Alzheimer’s disease [19]	NRF2 activation—omaveloxolone [16]; fumaric acid esters [23]
**2. Capacity exceeded**—in the output of the system as a whole	NRF2 translocates, and transcription is induced, but the response is not large enough	Response mounted, overwhelmed	Parkinson’s disease [19]	Burden reduction rather than response amplification. **No established class** [24,25]
**3. Constrained effector ceiling**—in the effector arm, downstream of NRF2	Regulator intact, but synthetic capacity is genetically limited downstream	Response mounted, capped	Schizophrenia, *GCLC* genotype [20]	Cysteine provision for glutathione synthesis—N-acetylcysteine [26]

**Table 2:** Conceptual development of the redox homeostasis model against the design of the trials that defined the field. Blank cells indicate that no corresponding event occurred in that column.

Year	Conceptual Milestone	Trial Design Implemented
1954–1956	Free radicals introduced as agents of injury and of aging [1,2]	—
1985	Pro-oxidant–antioxidant balance framed as the object of measurement in the intact cell or organ [9]	—
1991	Imbalance definition stated; causal versus associative relationship explicitly unresolved [10]	—
1993	—	DATATOP: α-tocopherol 2000 IU/day, fixed dose, no baseline or on-treatment redox measure [5]
1997	—	ADCS: α-tocopherol 2000 IU/day, fixed dose, no redox measure; significance contingent on post hoc covariate adjustment [6]
2006	Thiol/disulfide couples shown not to be mutually equilibrated; single global balance rejected; oxidative stress redefined as disruption of redox signaling and control [11]	—
2011	ROS established as physiological signaling intermediates acting through reversible cysteine modification [12]	—
2014	—	QE3: coenzyme Q10 at fixed dose, α-tocopherol 1200 IU/day in every arm including placebo, no redox measure; terminated for futility [7]. TEAM-AD: α-tocopherol 2000 IU/day, fixed dose, no redox measure; functional benefit on ADCS-ADL, no cognitive benefit [8]
2017	Concentration dependence formalized; eustress and distress ranges defined [13]	Edaravone: efficacy demonstrated only after enrollment restricted by stage, respiratory function, disease duration, and documented rate of decline [15]
2020	Unspecific scavenging judged ineffective; selective pathway targeting proposed [14]	—

**Table 3:** α-Tocopherol dosing in the principal neurodegeneration trials, set against doses at which adverse effects were detected in non-neurological populations. Multiples are calculated against the 400 IU/day threshold reported by Miller and colleagues [30]. ADCS, Alzheimer’s Disease Cooperative Study; TEAM-AD, Trial of Vitamin E and Memantine in Alzheimer’s Disease; SELECT, Selenium and Vitamin E Cancer Prevention Trial.

Study	Population	α-Tocopherol Dose (IU/Day)	Multiple of 400 IU/Day	Redox Measure at Entry
Miller et al. 2005 [30]	Pooled, 19 trials, n = 135,967	≥400 (harm threshold; risk emerging above 150)	1×	Not applicable
SELECT [31]	Cancer prevention, n = 35,533	400 (increased prostate cancer)	1×	None
DATATOP [5]	Early Parkinson’s disease, n = 800	2000	5×	None
ADCS [6]	Moderate Alzheimer’s disease, n = 341	2000	5×	None
TEAM-AD [8]	Mild to moderate Alzheimer’s disease, n = 613	2000	5×	None
QE3 [7]	Early Parkinson’s disease, n = 600	1200 (all arms, including placebo)	3×	None

**Table 4:** Assessment of currently available measurement classes against the requirements of a dysregulation model. “Partial” indicates that the requirement is met under conditions specified in the text. MDA, malondialdehyde; 8-OHdG, 8-hydroxy-2′-deoxyguanosine; MRS, magnetic resonance spectroscopy; NfL, neurofilament light chain; GFAP, glial fibrillary acidic protein; sTREM2, soluble triggering receptor expressed on myeloid cells 2; CNS, central nervous system.

Measurement Class	Reports Redox State, Not Damage	Reports CNS Compartment	Defines an Individual Baseline	Principal Limitation
Damage products (F2-isoprostanes, MDA, 8-OHdG, carbonyls)	No	No	Partial	Terminal products of oxidation; report damage already sustained, not regulatory state. Readings partially suppressed by NSAIDs (indomethacin, meclofenamic acid) [34]
Glutathione MRS	Yes	Yes	Partial	Resting concentration may not differ even where capacity does [36]; no established challenge protocol
Injury and glial markers (NfL, GFAP, YKL-40, sTREM2)	No	Yes	Yes	Not redox-specific. Report axonal injury and glial activation, which are downstream consequences of many processes; carry no information about redox regulation [28,29]
Redox-relevant genotype	No—reports capacity	Not compartment-specific	Yes (invariant)	Candidate-gene status; capacity is not current state [20,25]

**Table 5:** PROPOSED redox staging framework for neurological and psychiatric disease. Not derived from a published dataset; no thresholds are specified. Offered as a structural hypothesis requiring prospective validation in a single cohort. MRS, magnetic resonance spectroscopy; NfL, neurofilament light chain; GFAP, glial fibrillary acidic protein; sTREM2, soluble triggering receptor expressed on myeloid cells 2; AT(N), amyloid/tau/neurodegeneration biomarker classification.

Axis	What It Records	Candidate Measures	Status
**R—Reserve**	Capacity to defend a set-point under challenge, independent of resting values; determined by synthetic genotype together with hormonal status, developmental timing of insult, and accumulated environmental load	Redox-relevant genotype (*GCLC* and related) [20]. Proposed: recovery of the GSH/GSSG ratio in peripheral cells after a graded oxidant load, measured ex vivo (Section 3.3). No in vivo challenge measure has yet been established.	Genotype available; challenge protocol does not exist
**D—Displacement**	Whether a specific redox couple (e.g., GSH/GSSG) is currently outside its operating range, in which compartment and direction	Regional glutathione MRS [36]; compartment-specific thiol/disulfide couples	Measurable in principle; resting values may be uninformative [36]
**A—Activity**	Rate of change in the underlying process, measured over a defined observation period	Documented functional decline during run-in [15]; serial fluid markers	Demonstrated feasibility; the criterion that distinguished the successful trials
**N—Neurodegeneration**	Extent of downstream injury already sustained	NfL, GFAP, YKL-40, sTREM2 [28,29]; AT(N) where applicable [38]	Available and validated for staging position

## Data Availability

No new data were created or analyzed in this study. Data sharing is not applicable to this article.

## References

[1] Gerschman R., Gilbert D.L., Nye S.W., Dwyer P., Fenn W.O. (1954). Oxygen poisoning and x-irradiation: A mechanism in common. Science.

[2] Harman D. (1956). Aging: A theory based on free radical and radiation chemistry. J. Gerontol..

[3] Alam Z.I., Daniel S.E., Lees A.J., Marsden D.C., Jenner P., Halliwell B. (1997). A generalised increase in protein carbonyls in the brain in Parkinson’s but not incidental Lewy body disease. J. Neurochem..

[4] Alam Z.I., Halliwell B., Jenner P. (2000). No evidence for increased oxidative damage to lipids, proteins, or DNA in Huntington’s disease. J. Neurochem..

[5] The Parkinson Study Group (1993). Effects of tocopherol and deprenyl on the progression of disability in early Parkinson’s disease. N. Engl. J. Med..

[6] Sano M., Ernesto C., Thomas R.G., Klauber M.R., Schafer K., Grundman M., Woodbury P., Growdon J., Cotman C.W., Pfeiffer E. (1997). A controlled trial of selegiline, alpha-tocopherol, or both as treatment for Alzheimer’s disease. N. Engl. J. Med..

[7] Beal M.F., Oakes D., Shoulson I., Henchcliffe C., Galpern W.R., Haas R., Juncos J.L., Nutt J.G., Voss T.S., Ravina B. (2014). A randomized clinical trial of high-dosage coenzyme Q10 in early Parkinson disease: No evidence of benefit. JAMA Neurol..

[8] Dysken M.W., Sano M., Asthana S., Vertrees J.E., Pallaki M., Llorente M., Love S., Schellenberg G.D., McCarten J.R., Malphurs J. (2014). Effect of vitamin E and memantine on functional decline in Alzheimer disease: The TEAM-AD VA cooperative randomized trial. JAMA.

[9] Sies H., Cadenas E. (1985). Oxidative stress: Damage to intact cells and organs. Philos. Trans. R. Soc. Lond. B Biol. Sci..

[10] Sies H. (1991). Oxidative stress: From basic research to clinical application. Am. J. Med..

[11] Jones D.P. (2006). Redefining oxidative stress. Antioxid. Redox Signal..

[12] Finkel T. (2011). Signal transduction by reactive oxygen species. J. Cell Biol..

[13] Sies H. (2017). Hydrogen peroxide as a central redox signaling molecule in physiological oxidative stress: Oxidative eustress. Redox Biol..

[14] Sies H., Jones D.P. (2020). Reactive oxygen species (ROS) as pleiotropic physiological signaling agents. Nat. Rev. Mol. Cell Biol..

[15] Writing Group, Edaravone (MCI-186) ALS 19 Study Group (2017). Safety and efficacy of edaravone in well defined patients with amyotrophic lateral sclerosis: A randomised, double-blind, placebo-controlled trial. Lancet Neurol..

[16] Lynch D.R., Chin M.P., Delatycki M.B., Subramony S.H., Corti M., Hoyle J.C., Boesch S., Nachbauer W., Mariotti C., Mathews K.D. (2021). Safety and efficacy of omaveloxolone in Friedreich ataxia (MOXIe study). Ann. Neurol..

[17] Day B.J. (2026). Antioxidants in lung disease: Failures and pathways for success. Free Radic. Biol. Med..

[18] Dayalan Naidu S., Dinkova-Kostova A.T. (2020). KEAP1, a cysteine-based sensor and a drug target for the prevention and treatment of chronic disease. Open Biol..

[19] Ramsey C.P., Glass C.A., Montgomery M.B., Lindl K.A., Ritson G.P., Chia L.A., Hamilton R.L., Chu C.T., Jordan-Sciutto K.L. (2007). Expression of Nrf2 in neurodegenerative diseases. J. Neuropathol. Exp. Neurol..

[20] Gysin R., Kraftsik R., Sandell J., Bovet P., Chappuis C., Conus P., Deppen P., Preisig M., Ruiz V., Steullet P. (2007). Impaired glutathione synthesis in schizophrenia: Convergent genetic and functional evidence. Proc. Natl. Acad. Sci. USA.

[21] Solis-Paredes J.M., Montoya-Estrada A., Cruz-Rico A., Reyes-Muñoz E., Perez-Duran J., Espino Y Sosa S., Garcia-Salgado V.R., Sevilla-Montoya R., Martinez-Portilla R.J., Estrada-Gutierrez G. (2022). Plasma total antioxidant capacity and carbonylated proteins are increased in pregnant women with severe COVID-19. Viruses.

[22] Kocher M., McDermott M., Lindsey R., Shikuma C.M., Gerschenson M., Chow D.C., Kohorn L.B., Hetzler R.K., Kimura I.F. (2017). Short communication: HIV patient systemic mitochondrial respiration improves with exercise. AIDS Res. Hum. Retroviruses.

[23] Freudenstein D., Schneeweiss A., Krenz A., Gold R., Linker R., Haase S. (2026). Diroximel fumarate confers neuroprotection via reduced Th1 responses and induction of the anti-oxidative Nrf2 pathway in experimental neuroinflammation. PLoS ONE.

[24] Snow B.J., Rolfe F.L., Lockhart M.M., Frampton C.M., O’Sullivan J.D., Fung V., Smith R.A.J., Murphy M.P., Taylor K.M. (2010). A double-blind, placebo-controlled study to assess the mitochondria-targeted antioxidant MitoQ as a disease-modifying therapy in Parkinson’s disease. Mov. Disord..

[25] Karaa A., Bertini E., Carelli V., Cohen B.H., Enns G.M., Falk M.J., Goldstein A., Gorman G.S., Haas R., Hirano M. (2023). Efficacy and safety of elamipretide in individuals with primary mitochondrial myopathy: The MMPOWER-3 randomized clinical trial. Neurology.

[26] Rossell S.L., Francis P.S., Galletly C., Harris A., Siskind D., Berk M., Bozaoglu K., Dark F., Dean O., Liu D. (2016). N-acetylcysteine (NAC) in schizophrenia resistant to clozapine: A double blind randomised placebo controlled trial targeting negative symptoms. BMC Psychiatry.

[27] Berendse H.W., Booij J., Francot C.M., Bergmans P.L., Hijman R., Stoof J.C., Wolters E.C. (2001). Subclinical dopaminergic dysfunction in asymptomatic Parkinson’s disease patients’ relatives with a decreased sense of smell. Ann. Neurol..

[28] Pelkmans W., Shekari M., Brugulat-Serrat A., Sánchez-Benavides G., Minguillón C., Fauria K., Molinuevo J.L., Grau-Rivera O., González Escalante A., Kollmorgen G. (2024). Astrocyte biomarkers GFAP and YKL-40 mediate early Alzheimer’s disease progression. Alzheimer’s Dement..

[29] Nordengen K., Kirsebom B.E., Henjum K., Selnes P., Gísladóttir B., Wettergreen M., Torsetnes S.B., Grøntvedt G.R., Waterloo K.K., Aarsland D. (2019). Glial activation and inflammation along the Alzheimer’s disease continuum. J. Neuroinflamm..

[30] Miller E.R., Pastor-Barriuso R., Dalal D., Riemersma R.A., Appel L.J., Guallar E. (2005). Meta-analysis: High-dosage vitamin E supplementation may increase all-cause mortality. Ann. Intern. Med..

[31] Klein E.A., Thompson I.M., Tangen C.M., Crowley J.J., Lucia M.S., Goodman P.J., Minasian L.M., Ford L.G., Parnes H.L., Gaziano J.M. (2011). Vitamin E and the risk of prostate cancer: The Selenium and Vitamin E Cancer Prevention Trial (SELECT). JAMA.

[32] Upston J.M., Terentis A.C., Stocker R. (1999). Tocopherol-mediated peroxidation of lipoproteins: Implications for vitamin E as a potential antiatherogenic supplement. FASEB J..

[33] Bowry V.W., Mohr D., Cleary J., Stocker R. (1995). Prevention of tocopherol-mediated peroxidation in ubiquinol-10-free human low density lipoprotein. J. Biol. Chem..

[34] Kadiiska M.B., Gladen B.C., Baird D.D., Graham L.B., Parker C.E., Ames B.N., Basu S., FitzGerald G.A., Lawson J.A., Marnett L.J. (2005). Biomarkers of oxidative stress study III. Effects of the nonsteroidal anti-inflammatory agents indomethacin and meclofenamic acid on measurements of oxidative products of lipids in CCl4 poisoning. Free Radic. Biol. Med..

[35] Karaa A., Haas R., Goldstein A., Vockley J., Cohen B.H. (2020). A randomized crossover trial of elamipretide in adults with primary mitochondrial myopathy. J. Cachexia Sarcopenia Muscle.

[36] Girgis R.R., Baker S., Mao X., Gil R., Javitt D.C., Kantrowitz J.T., Gu M., Spielman D.M., Ojeil N., Xu X. (2019). Effects of acute N-acetylcysteine challenge on cortical glutathione and glutamate in schizophrenia: A pilot in vivo proton magnetic resonance spectroscopy study. Psychiatry Res..

[37] McGorry P.D., Hickie I.B., Yung A.R., Pantelis C., Jackson H.J. (2006). Clinical staging of psychiatric disorders: A heuristic framework for choosing earlier, safer and more effective interventions. Aust. N. Z. J. Psychiatry.

[38] Jack C.R., Bennett D.A., Blennow K., Carrillo M.C., Dunn B., Haeberlein S.B., Holtzman D.M., Jagust W., Jessen F., Karlawish J. (2018). NIA-AA Research Framework: Toward a biological definition of Alzheimer’s disease. Alzheimer’s Dement..

[39] Amariglio R.E., Grill J.D., Rentz D.M., Marshall G.A., Donohue M.C., Liu A., Aisen P.S., Sperling R.A. (2024). Longitudinal trajectories of the Cognitive Function Index in the A4 Study. J. Prev. Alzheimer’s Dis..

[40] Garcia-Rizo C., Fernandez-Egea E., Oliveira C., Meseguer A., Cabrera B., Mezquida G., Bioque M., Penades R., Parellada E., Bernardo M. (2017). Metabolic syndrome or glucose challenge in first episode of psychosis?. Eur. Psychiatry.

